# Heterozygous *Dcc* Mutant Mice Have a Subtle Locomotor Phenotype

**DOI:** 10.1523/ENEURO.0216-18.2021

**Published:** 2022-03-02

**Authors:** Louise Thiry, Chloé Lemaire, Ali Rastqar, Maxime Lemieux, Jimmy Peng, Julien Ferent, Marie Roussel, Eric Beaumont, James P. Fawcett, Robert M. Brownstone, Frédéric Charron, Frédéric Bretzner

**Affiliations:** 1 Centre de Recherche du Centre Hospitalier Universitaire (CHU) de Québec-Université Laval, Centre Hospitalier de l’Université Laval (CHUL)–Neurosciences P09800, Quebec City, Quebec G1V 4G2, Canada; 2 Institut de Recherches Cliniques de Montréal (IRCM), Montréal, Quebec H2W 1R7, Canada; 3 Department of Biology, McGill University, Montréal, Quebec H3G 0B1, Canada; 4 Department of Biomedical Sciences, Quillen College of Medicine, East Tennessee State University, Johnson City, TN 37604; 5 Department of Pharmacology, Dalhousie University, Halifax, Nova Scotia B3H 4R2, Canada; 6 Department of Surgery, Dalhousie University, Halifax, Nova Scotia B3H 4R2, Canada; 7 University College London (UCL) Queen Square Institute of Neurology, University College London, London WC1N 3BG, United Kingdom; 8 Department of Medicine, University of Montreal, Montréal, Quebec H3C 3J7, Canada; 9 Department of Psychiatry and Neurosciences, Université Laval, Quebec City, Quebec G1V 4G2, Canada

**Keywords:** CPG, Dcc, locomotion, mutant mice

## Abstract

Axon guidance receptors such as deleted in colorectal cancer (DCC) contribute to the normal formation of neural circuits, and their mutations can be associated with neural defects. In humans, heterozygous mutations in *DCC* have been linked to congenital mirror movements, which are involuntary movements on one side of the body that mirror voluntary movements of the opposite side. In mice, obvious hopping phenotypes have been reported for bi-allelic *Dcc* mutations, while heterozygous mutants have not been closely examined. We hypothesized that a detailed characterization of *Dcc* heterozygous mice may reveal impaired corticospinal and spinal functions. Anterograde tracing of the *Dcc*^+/−^ motor cortex revealed a normally projecting corticospinal tract, intracortical microstimulation (ICMS) evoked normal contralateral motor responses, and behavioral tests showed normal skilled forelimb coordination. Gait analyses also showed a normal locomotor pattern and rhythm in adult *Dcc*^+/−^ mice during treadmill locomotion, except for a decreased occurrence of out-of-phase walk and an increased duty cycle of the stance phase at slow walking speed. Neonatal isolated *Dcc*^+/−^ spinal cords had normal left-right and flexor-extensor coupling, along with normal locomotor pattern and rhythm, except for an increase in the flexor-related motoneuronal output. Although *Dcc*^+/−^ mice do not exhibit any obvious bilateral impairments like those in humans, they exhibit subtle motor deficits during neonatal and adult locomotion.

## Significance Statement

We show that loss of one deleted in colorectal cancer (*Dcc*) allele does not affect motor cortex, corticospinal efficacy, or skilled locomotor control in adult mice, but it increases flexor-related motoneuronal output in the developing spinal cord and increases duty cycle of the stance phase during treadmill locomotion at slow walking speeds in adult mice. This finding raises the possibility of the existence of subtle locomotor changes in humans carrying monoallelic *DCC* mutations.

## Introduction

Individuals carrying monoallelic deleted in colorectal cancer (*DCC*) mutations exhibit congenital mirror movements, which are unintentional movements on one side of the body that are mirror reversals of intended unilateral movements on the opposite side (Schott and Wyke, 1981; Srour et al., 2010). Bilateral motor responses can be evoked in response to unilateral transcranial magnetic stimulation of the motor cortex in these people (Srour et al., 2010; Welniarz et al., 2017) and, these bilateral motor responses are correlated with an abnormal bilateral projection of their corticospinal fibers at the level of the pyramidal decussation (Welniarz et al., 2017). This phenotype in *DCC* heterozygous humans is most likely a result of haplo-insufficiency, given that many of the mutations are predicted to result in mRNA degradation or truncated DCC proteins (Izzi and Charron, 2011; Peng and Charron, 2013). The receptor DCC mediates a chemoattractive signal to Netrin-1 (Keino-Masu et al., 1996; Fazeli et al., 1997; Jain et al., 2014; Srivatsa et al., 2014), thereby contributing to the normal development of a wide variety of axonal tracts, including those of spinal commissural interneurons (Rabe Bernhardt et al., 2012) and corticospinal tracts (Finger et al., 2002). Mutations in *NETRIN-1* also cause mirror movements in human (Méneret et al., 2017). Loss of floor plate Netrin-1 in mice impairs midline crossing of corticospinal and spinal axons and leads to a bilateral forelimb movement phenotype reminiscent of human mirror movements (Pourchet et al., 2021).

In contrast to *DCC*^+/−^ humans, heterozygous *Dcc*^+/−^ mice exhibit no overt mirror-like phenotypes. Bi-allelic *Dcc* null mutant mice die at birth, precluding the possibility of studying their motor cortex and corticospinal tract projection through adulthood (Fazeli et al., 1997). However, mutant mice carrying the bi-allelic *kanga* mutation (*Dcc^kanga^*^/kanga^), a spontaneous mutation that removes the P3 intracellular domain, are viable and exhibit an abnormal rabbit-like hopping gait (Finger et al., 2002). The hopping gait phenotype is different from the bilateral forelimb movement phenotype: while the former is mostly known to occur because of axon crossing defects in spinal interneurons (Kullander et al., 2003; Talpalar et al., 2013; Peng et al., 2018), the latter has been linked to crossing defects of the corticospinal tract (Serradj et al., 2014; Pourchet et al., 2021). Corticospinal tract lateralization defects produce phenotypes more akin to the mirror-movement phenotype observed in *DCC*^+/−^ humans.

DCC and its ligand Netrin-1 are also important for normal development of sensory afferents to the dorsal spinal cord (Ding et al., 2005; Watanabe et al., 2006) and spinal commissural interneurons (Keino-Masu et al., 1996; Fazeli et al., 1997; Rabe et al., 2009; Rabe Bernhardt et al., 2012; Dominici et al., 2017; Varadarajan et al., 2017). Spinal cords isolated from neonatal wild-type (WT) mice produce spontaneous left-right alternating neuronal activity on bath application of neurotransmitters, which reflect the output of the locomotor central pattern generator (Jiang et al., 1999; Thiry et al., 2016). Interestingly, neonatal *Netrin-1* mutant spinal cords exhibit a reduction in the number of commissural interneurons including V0 commissural interneurons, whereas V3 commissural interneurons are spared and presumably contribute to the synchronization of left and right locomotor activities (Rabe Bernhardt et al., 2012). However, both neonatal *Dcc*^−/−^ and *Dcc*^kanga/kanga^ spinal cords exhibit a robust reduction in the number of most commissural interneurons, including V0 and V3 commissural interneurons, thus leading to disorganization of the coupling between left and right locomotor activities (Rabe Bernhardt et al., 2012). More recently, it has been shown that a selective *Dcc* mutation in spinal interneurons (*HoxB8^cre^; Dcc^flox/−^* and *HoxB8^cre^; Dcc^flox/flox^*) exhibits a robust hopping phenotype in adult mice (Peng et al., 2018), indicating that local spinal cord defects following loss of *Dcc* cause a hopping gait. Given the strong phenotype reported in various neonatal and adult biallelic *Dcc* mutant spinal cords, we hypothesized that a heterozygous *Dcc* mutation might be sufficient to result in a neuroanatomical, neurophysiological, and motor phenotype, aiding in our understanding of the impairment of motor control seen in people carrying monoallelic *DCC* mutations.

Using axonal tract tracing, intracortical microstimulation (ICMS), and behavioral tests, we found that pyramidal decussation was normal, as was corticospinal efficacy in producing responses in forelimb and hindlimb muscles of adult *Dcc*^+/−^ mice, with no obvious functional impairments in their skilled motor control. Furthermore, no gait and posture dysfunctions were observed during treadmill locomotion, except for a decrease in the occurrence of out-of-phase walk and a longer duty cycle for the stance phase at slow treadmill speed. In spinal cord preparations isolated from neonatal mice, spinal interneuronal circuits exhibited normal locomotor pattern and rhythm; nevertheless, the flexor-related motoneuronal output was significantly increased in neonatal *Dcc*^+/−^ spinal cords. In summary, although *Dcc*^+/−^ mice do not exhibit any obvious bilateral impairments like those in humans, they exhibit subtle motor deficits during neonatal and adult locomotion.

## Materials and Methods

All animal procedures were performed in accordance with the Dalhousie University, Institut de Recherches Cliniques de Montréal (IRCM) and Université Laval animal care committee’s regulations. *Dcc*^+/−^ mice were previously generated by the insertion of a neomycin resistance cassette into exon three of the *Dcc* gene (Fazeli et al., 1997). Immunoprecipitation experiments demonstrated that no full-length protein was produced from this allele in homozygous mutant mice (Fazeli et al., 1997).

### DCC protein level quantification by Western blotting

Spinal cords were dissected at E13.5, similarly to previously published (Langlois et al., 2010). Tissue was lysed with RIPA buffer (50 mm HEPES pH 7.4, 150 mm NaCl, 10% glycerol, 1.5 mm MgCl_2_, 1% Triton X-100, 1% SDS, and 1 mm EDTA) with protease inhibitors (Roche 11873580001) and boiled in SDS sample buffer for 5 min. Protein samples were separated by SDS-PAGE and then transferred to PVDF membrane. The membranes were incubated with 5% skim milk in TBST (0.01 m Tris-HCl pH 7.5, 150 mm NaCl, and 0.1% Tween 20) for 1 h at room temperature, followed by primary antibody incubation (goat anti-DCC,1:400, A-20, Santa Cruz Biotechnology and mouse anti-actin, 1:1000, Sigma, catalog #A5441) in 1% skim milk in TBST, overnight at 4°C. After three washes in TBST, membranes were incubated for 2 h at room temperature, with secondary antibodies, which were conjugated to horseradish peroxidase (anti-goat HRP, 1:10,000, Jackson ImmunoResearch, catalog #705-035-147 and anti-mouse-HRP, 1:10,000, Jackson ImmunoResearch, catalog #115-035-003). After three washes in TBST and a final wash in TBS (without Tween 20), Western blottings were visualized with chemiluminescence.

### BDA tracing and analysis of the corticospinal tract

Five adult WT and five *Dcc*^+/−^ animals were anesthetized with ketamine/xylazine (100/10 mg/kg body weight) and a hand drill (Dremel) was used to create a small opening in the skull; 5 μl of biotinylated dextran amine (10% in PBS, Invitrogen, 10,000 MW) was injected unilaterally into the motor cortex with a syringe (Hamilton, 80 300) and the animal was sutured and allowed to recover. After 14 d, animals were perfused in 4% paraformaldehyde (PFA) in PBS and the spinal cord and brain were dissected and postfixed in 4% PFA overnight before cryoprotection in 30% sucrose in PBS and freezing of segments in tissue freezing medium (O.C.T. compound); 30-μm cryosections of brain and spinal cord were incubated in streptavidin-488 (Jackson ImmunoResearch, 1:200 in PBS + 0.1% Triton X-100) for 2 h at room temperature, mounted, and imaged with a Leica DM4000 fluorescent microscope.

### ICMS

Mice were anaesthetized with ketamine-xylazine (100/10 mg/kg body weight). When necessary, supplementary doses of ketamine were administered. The cranial bone was drilled to expose the motor cortex (approximate coordinates bregma +2 to −3, lateral 0.5–3). A tungsten electrode (0.1 MΩ) was inserted up to a depth of 0.7–0.8 mm. Cathodal pulses (10–80 μA, 0.2-ms duration, trains of 30 ms, interval 2.8 ms) were delivered through this electrode. A silver wire attached to the skin was used as the anode. To evoke motor response in the hindlimb, the electrode was positioned in the hindlimb representation of the motor cortex (about bregma −1 to −2 mm, lateral −1 to −2) in 11 WT mice and 13 *Dcc*^+/−^ mice. In five WT mice and five *Dcc*^+/−^ mice, we stimulated the forelimb caudal areas (bregma 0 to −1, lateral −1 to −2).

Electromyographic (EMG) probes organized in a duplex configuration (Ritter et al., 2014; Lemieux and Bretzner, 2019) were inserted in the tibialis anterior (TA) on both sides. When we stimulated the forelimb caudal area, we inserted EMG probes in the biceps brachialis (BB). For technical reason, we did not attempt to record the ipsilateral BB. The threshold was evaluated to evoke movements of the ankle and/or knee for the hindlimb and the wrist and/or elbow for the forelimb. The threshold was defined as the current intensity evoking movements 50% of the time or more. For EMG recordings, success rates, latencies, and the number of motor spikes were quantified. We analyzed the number of motor spikes rather than the amplitude of motor spikes because the number of spikes is less dependent on the position of electrodes, which makes it a more reliable approximation of the motor response.

### Skilled motor and locomotor behaviors

#### Cylinder test

Unilateral and bilateral forelimb movements were assessed in a glass beaker for the cylinder test, a vertical exploratory test (Bretzner et al., 2008, 2010; Sparling et al., 2015). A mirror was placed behind the cylinder with an angle to have an overall view of the mouse. Mice were videotaped for 20 rightings using a 40-Hz camera. The use of the left, right, or both forelimbs was scored as the first paw contact and the total number of contacts for each righting. To evaluate motor lateralization, the score was expressed as a percentage of use of the left, right, or both forelimbs relative to the total number of first or total forepaw contacts.

#### Beam locomotion

Motor coordination and balance were assessed while mice crossed a wide (12 mm width) and a narrow (6 mm width) beam of 40 cm long each (Luong et al., 2011; Fleming et al., 2013). After training, mice were videotaped at a sampling frequency of 40 Hz for three crossings. The number of steps, foot-slip errors, and the time to cross the beam were quantified offline from videos. The percentage of foot-slips was computed as the number of foot-slip errors relative of the number of steps for each crossing and was then averaged for three trials per animal.

#### Horizontal ladder locomotion

Mice were trained to walk on a horizontal ladder with a regular (1 cm spacing) rung arrangement pattern (Metz and Whishaw, 2002; Laflamme et al., 2019). After training, mice were videotaped for three crossings using a 40-Hz camera. Videos were analyzed frame by frame to assess the number of steps, foot-slip errors, and the time to cross the ladder. The percentage of foot-slip errors was calculated as the number of errors relative of the number of steps for each trial. The number of steps and the percentage of foot-slip errors were averaged for each mouse for the three trials. The percentage of hindpaw slipping was not reported because it happened only when mice fell from the ladder after forepaw slip.

### Treadmill locomotion

Eight WT and nine *Dcc*^+/−^ six-month-old mice were placed on a treadmill (Cleversys Systems Inc.) equipped with a transparent belt. The treadmill speed was adjusted at 15, 20, and 30 cm/s. Each mouse performed two 20-s trials at each speed (from lowest to highest) and was allowed a 3-min rest between each trial. All mice were filmed from below the belt with a high-frequency camera (100 frames/s, Basler) and videos were analyzed offline using custom software as previously described (Lemieux et al., 2016). To avoid acceleration and deceleration phases, videos were analyzed during steady-state locomotion. The timing of lifts and contacts for all four limbs were extracted manually and used for step cycle analysis by computing (1) stance duration: the interval between the foot contact with the belt and the subsequent foot lift; (2) swing duration: the interval between the foot lift and the next foot contact; (3) step cycle: the interval between two successive foot contacts in each limb; and (4) stride frequency: the inverse of the step cycle.

### Gait analysis during treadmill locomotion

Locomotor gaits were defined by the interlimb coupling between stance phases of four limbs and locomotor frequencies (number of step cycles per second). Using custom-written routines in MATLAB (The MathWorks), gaits of WT and *Dcc*^+/−^ mice were analyzed during treadmill locomotion at 15, 20, and 30 cm/s. As these speeds were low to intermediate, analysis was focused on three gaits: out-of-phase walk, lateral walk, and trot (Lemieux et al., 2016). Two slow walking gaits, pace and diagonal walk, were excluded from the analysis because of their weak occurrence in mice.

### Neonatal locomotor-like activity

Spinal cords from WT and *Dcc*^+/−^ mice dissected out on postnatal days 1–3 were used for *in vitro* experiments. Animals were anesthetized by intraperitoneal injection of ketamine/xylazine (100/10 mg/kg), decapitated, and eviscerated. Spinal cords were isolated by vertebrectomy at room temperature in oxygenated (95% O_2_, 5% CO_2_) artificial CSF (aCSF) containing 127 mm NaCl, 3 mm KCl, 26 mm NaHCO_3_, 1.25 mm NaH_2_PO_4_, 2 mm CaCl_2_, 1 mm MgCl_2_, and 10 mm glucose. Spinal cords were cut at the thoracic Th10/11 and sacral S2/3 levels and placed ventral side up in a recording chamber superfused with oxygenated aCSF. Left and right lumbar L2 and L5 ventral roots were attached to suction electrodes designed and selected to fit the specific size of each recorded ventral root, thus ensuring a perfect seal of the suction electrode. The spinal cord was then allowed to recover for at least 30 min before electroneurographic (ENG) recording. Chemically evoked locomotor-like activity was induced by bath application of a cocktail of neurotransmitters: 5-hydrotryptamine (5HT; 10 μm; Abcam) and an increased concentration of NMDA (2.5, 5, and 7.5 μm; Fisher) for episodes of ∼30 min at each concentration. The signals were amplified (gain 2000) and bandpass filtered 10 Hz to 5 kHz (Qi-Ying Design). Signals were sampled at 50 kHz (Digidata 1440A, Molecular Devices) and stored on a PC (Axoscope 10.3; Molecular Device) for offline analysis. The amplitude and duration of ENG bursts, the stride duration, the interburst duration, the duty cycle, and the coupling were analyzed on a 300-s epoch (25–60 locomotor cycles) of locomotor-like activity using Spinalcore. The amplitude was measured from the baseline (0) of integrated signals.

### Statistics

Data for male and female mice were pooled together. Visual inspection suggested that the data were similar between sexes. Circular statistics was used to calculate the robustness of phase couplings between limbs or ENGs during locomotion; Rayleigh values are illustrated as the distance from the center of the polar plot (Drew and Doucet, 1991; Kjaerulff and Kiehn, 1996; Zar, 1996). A phase of 0 (or 1) indicates synchronization, whereas a phase of 0.5 corresponds to an alternation. The statistical significance of phase differences between WT and *Dcc*^+/−^ mice was tested with a Watson–William test. Error bars shown are mean ± SD of the average or the coefficient of variation (CV). The normality of the distribution was assessed with Shapiro–Wilk before two-sample testing. Before pooling data, we tested the homogeneity of variances with a Fisher (two-samples, left and right limb) or Bartlett test (multiple samples, beam and horizontal ladder crossing). To detect differences between the mouse genotypes during adult treadmill locomotion, neonatal locomotor-like activity, and in anatomic measurements, we used the *t* test or nonparametric Mann–Whitney ranked sum test when the variables did not fit a normal distribution (assessed by Kolmogorov–Smirnov test).

## Results

### Skilled motor control and locomotion in *Dcc*^+/−^ adult mice

**Figure 1.**
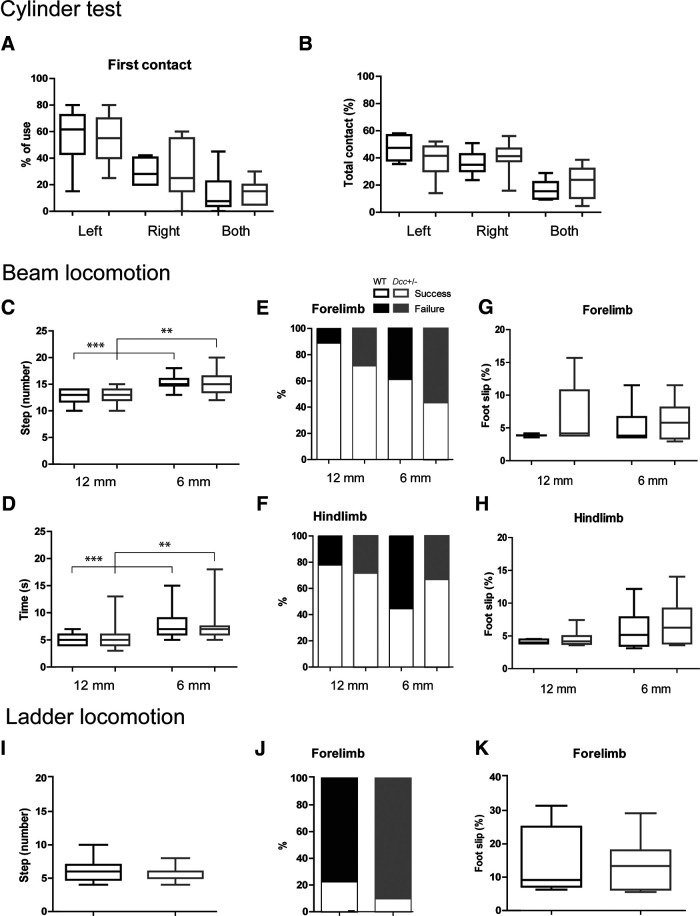
Skilled motor control in adult *Dcc*^+/−^ and WT mice. ***A***, ***B***, Percentage of first and total contacts on the wall while rearing in the cylinder test. ***C–H***, Mean number of steps (***C***), time (***D***), percentage of successful and failed trials with the forelimb (***E***) and hindlimb (***F***), percentage of foot slips with the forelimb (***G***) and hindlimb (***H***) among trials with errors. ***I–K***, Mean number of steps (***I***), percentage of successful and failed trials (***J***), and percentage of foot slips among failed trials (***K***) during locomotion on the rungs of a horizontal ladder. WT in black and *Dcc*^+/−^ in gray; *p < 0.05, **p < 0.01, and ***p < 0.001 (for statistics, see Extended Data Table 1-1).

In mice, rats, and cats, while locomotion on a smooth horizontal surface may rely solely on subcortical and spinal motor systems (Z’Graggen et al., 1998; Soblosky et al., 2001), the motor cortex plays an important role in the control of voluntary motor tasks such as skilled forelimb reaching (Whishaw, 2000; Bretzner et al., 2008, 2010; Sparling et al., 2015), as well as skilled locomotion while the animal has to adjust its precise paw and limb trajectory to avoid obstacles (Drew et al., 1996; Metz and Whishaw, 2002; Friel et al., 2007; Laflamme et al., 2019). Previous work showed that Dcc protein levels are reduced in the brain of adult *Dcc*^+/−^ mice compared with WT mice (Flores et al., 2005). Using a test of vertical exploration to assess skilled motor control (Fig. 1*A*,*B*), we quantified the percentage of initial and subsequent use of left, right, or both forepaws while reaching the wall of a cylinder during rearing. In comparison to control WT mice, the *Dcc*^+/−^ mice displayed no differences in the percentage of use of their left, right, or both forepaws in the cylinder during the first contact on the wall (*n* = 6 WT and 7 *Dcc*^+/−^ mice, Mann–Whitney test for left forepaw use, *p* = 0.9226; right forepaw, *p* = 0.6669; and both forepaws, *p* = 0.5163; Fig. 1*A*), and during the total number of contacts (Mann–Whitney test for total individual use of the left forepaw, *p* = 0.2343; the right forepaw, *p* = 0.5643; and both forepaws, *p* = 0.3534; Fig. 1*B*), thus suggesting a normal use of forelimbs during vertical exploration.

10.1523/ENEURO.0216-18.2021.t1-1 Extended Data Table 1-1 Skilled motor control in adult *Dcc*^+/−^ and WT mice. Download Table 1-1, DOC file.

To assess balance and motor coordination, we evaluated mice while they walked on a horizontal wide (12-mm width) or narrow (6-mm width) beam (Fig. 1*C–H*). We first assessed whether there was a learning effect on our measurements on three beam crossings. As there were no significant effects on the number of steps, the time, and the percentage of foot-slips between the three crossings, data were pooled (Fig. 1*C*,*D*,*G*,*H*, Bartlett or Fischer test, *p* > 0.05; for statistics, see Extended Data Table 1-1). Although both WT and *Dcc*^+/−^ mice took more steps to cross the narrow beam than the wide one (*n* = 6 × 3 WT and 7 × 3 *Dcc*^+/−^ crossings, Mann–Whitney test, *p* = 0.0001 for WT and *p* = 0.0011 for *Dcc*^+/−^ on the 6- vs 12-mm beam; Fig. 1*C*), no statistical differences were observed according to genotype (Mann–Whitney test, *p* = 0.8392 for WT vs *Dcc*^+/−^ on the 6-mm beam; and *p* = 0.3193 for WT vs *Dcc*^+/−^ on the 12-mm beam; Fig. 1*C*). Similarly, the time to cross the wide beam was significantly shorter than to cross the narrow beam (Mann–Whitney test, *p* = 0.0002 for WT and *p* = 0.0011 for *Dcc*^+/−^ on the 6- vs 12-mm beam; Fig. 1*D*); nevertheless, no differences were found according to genotype (Mann–Whitney test, *p* = 0.6145 for WT vs *Dcc*^+/−^ on the 6-mm beam; *p* = 0.8249 for WT vs *Dcc*^+/−^ on the 12-mm beam; Fig. 1*D*). The proportion of successful/failed crossings was not significantly different while crossing the wide or the narrow beam with the forelimbs (Mann–Whitney test, *p* = 0.1398 for the WT forelimbs and *p* = 0.1626 for the *Dcc*^+/−^ forelimbs on the 6- vs 12-mm beam; Fig. 1*E*) or the hindlimbs (Mann–Whitney test *p* = 0.1534 for the WT hindlimbs and *p* = 0.9456 for the *Dcc*^+/−^ hindlimbs on the 6- vs 12-mm beam; Fig. 1*F*). Among the failed crossings, the percentage of foot-slips was not significantly different with the forelimbs on the narrow beam (*n* = 7/18 WT and 12/21 *Dcc*^+/−^ crossings, Mann–Whitney test, *p* = 0.8322; Fig. 1*G*) or the hindlimbs on the narrow or wide beam (*n* = 10/18 WT and 7/21 *Dcc*^+/−^ crossings on the narrow beam, Mann–Whitney test, *p* = 0.4336; *n* = 4/18 WT and 6/21 *Dcc*^+/−^ crossings on the wide beam, Mann–Whitney test, *p* = 1; Fig. 1*H*) according to mouse genotype, thus supporting a proper locomotor balance and motor coordination in *Dcc*^+/−^ mice.

To evaluate whether skilled forelimb locomotion might be impaired on *Dcc* mutation, we also assessed mice while crossing a horizontal ladder with even-spaced rungs, a situation where there is a need for precise limb trajectories and paw placements. (Fig. 1*I–K*). As with beam locomotion, we assessed whether there was a learning effect over the subsequent crossings. As there was no significant effect on the number of steps and the percentage of foot-slips over three crossings (Fig. 1*I*,*J*, Bartlett test, *p* > 0.05; for statistics, see Extended Data Table 1-1), data were pooled. Both WT and *Dcc*^+/−^ mice exhibited no statistical differences in the number of steps and in the percentage of foot-slips while walking on the rungs of the horizontal ladder (Mann–Whitney test for the number of steps of *n* = 18 WT vs 21 *Dcc*^+/−^ crossings, *p* = 0.8840; Mann–Whitney test for the proportion of successful vs failed crossings, *p* = 0.6614; Fig. 1*I*). Among failed crossings, the percentage of foot-slips with the forelimb was also not significantly different according to genotype (Mann–Whitney test, *p* = 0.4091; Fig. 1*K*). Taken together, *Dcc*^+/−^ mice display normal posture and balance overall, as well as normal skilled forelimb coordination and placement during skilled locomotion on a beam or a ladder.

### Anatomy of the corticospinal tract in the adult mouse

**Figure 2.**
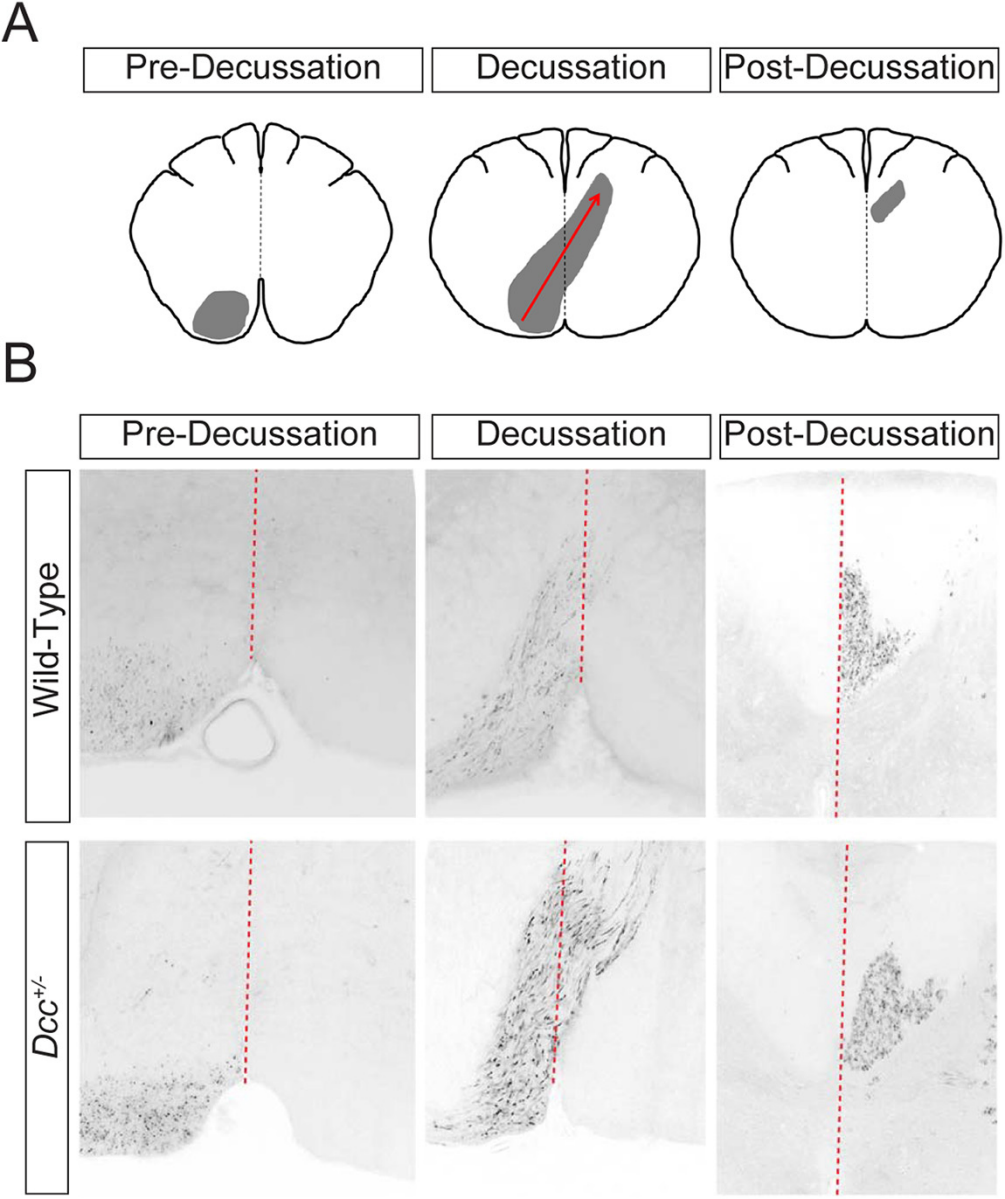
Projections of the corticospinal tract in *Dcc*^+/−^ and WT mice. ***A***, Schematic drawing of transverse brainstem sections showing a unilateral corticospinal tract axon bundle (gray area) as it projects from the left motor cortex to the contralateral dorsal funiculus. ***B***, BDA tracing of the corticospinal tract of three-month-old WT and *Dcc*^+/−^ mice shows no difference in the projection of corticospinal tract axons at the level of the pyramidal decussation.

A single-allele *Dcc* mutation is sufficient to alter pyramidal decussation and induce an aberrant bilateral misprojection of the corticospinal tract in humans (Srour et al., 2010; Welniarz et al., 2017). Thus, we asked whether a heterozygous *Dcc* mutation in mice might also be sufficient to impair normal projection of the corticospinal tract. As shown in Figure 2, axonal tract tracing of the motor cortex revealed that the projection of corticospinal axons at the level of the pyramidal decussation (Fig. 2*B*, middle panels) or postdecussation were similar in both *Dcc*^+/−^ and WT mice (Fig. 2*B*, right-most panels, five adult WT and five *Dcc*^+/−^ animals), suggesting that the corticospinal tract projects normally in adult *Dcc*^+/−^ mice. These results are consistent with previous observations (Welniarz et al., 2017).

### Functional connectivity of the corticospinal tract in the adult mouse

**Figure 3.**
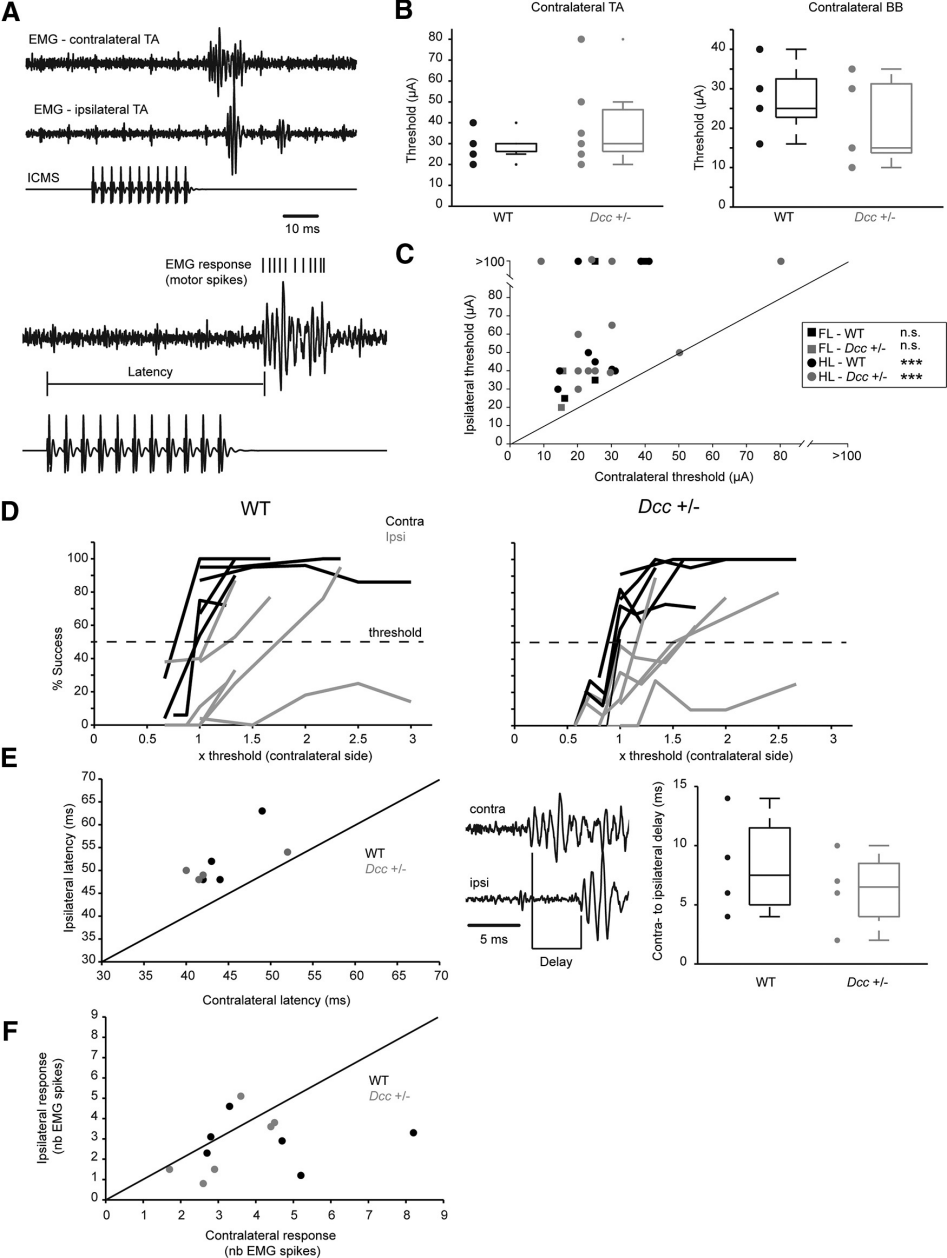
*Dcc*^+/−^ adult mice exhibit normal lateralization of the corticospinal tract. ***A***, Examples of EMG activity of contralateral and ipsilateral TA. A 30-ms train of cathodal pulses (duration 0.2 ms, interval 2.8 ms) was delivered in either the caudal forelimb area or the hindlimb area. Bottom, Higher temporal resolution of the contralateral trace illustrates latency and response (motor spikes raster) measurements. ***B***, left, Threshold for evoking activity in the contralateral BB (BB). Right, Threshold for evoking activity in the contralateral TA. Threshold is defined as motor spikes elicited in at least 50% of trials. ***C***, Thresholds for pairs of hindlimbs (HL; circle) or forelimbs (FL; square). Contralateral is on the *x*-axis and ipsilateral on the *y*-axis. ***D***, Success rate (percentage) for evoking an EMG response versus the threshold of the contralateral side. Dashed line indicates the threshold, defined as a success rate of 50%. Data are for pairs of muscle recorded with EMGs. Contralateral is in black and ipsilateral in gray. ***E***, left, Ipsilateral versus contralateral averaged latencies for pairs of muscles recorded with EMGs. Middle, An example of EMG traces to illustrate the delay between contralateral and ipsilateral sides. Right, Boxplot of contralateral to ipsilateral delays. ***F***, Averaged number of motor spikes evoked by ICMS for the contralateral (*x*-axis) and ipsilateral (*y*-axis) sides. WT in black and *Dcc*^+/−^ in gray; *** *P* < 0.001.

While our anatomic studies revealed that corticospinal tract projection appears normal in *Dcc*^+/−^ mice, the cortical representation and/or functional connectivity could still be impaired and evoke aberrant bilateral or ipsilateral movements in both forelimb and hindlimb muscles. To test this hypothesis, we recorded motor and EMG responses from bilateral forelimb and hindlimb muscles evoked by ICMS in the cortical caudal forelimb and hindlimb areas of adult WT and *Dcc*^+/−^ adult mice. As shown in Figure 3*A*, ICMS applied within the cortical representation of the hindlimb evoked the strongest EMG responses in the contralateral hindlimb muscle, TA, and weaker responses in the ipsilateral TA muscle of WT and *Dcc*^+/−^ mice at supra-threshold. To assess changes in corticospinal efficacy, we compared the threshold of cortically evoked motor responses in contralateral *Dcc*^+/−^ and WT forelimb BB and hindlimb TA muscles. Overall, there was no difference in the thresholds for evoking EMG responses in contralateral WT and *Dcc*^+/−^ forelimb and hindlimb muscles (Mann–Whitney test: *n* = 7 WT and 6 *Dcc*^+/−^ for the TA, *p* = 0.50; and *n* = 5 WT and 5 *Dcc*^+/−^ for the BB, *p* = 0.34; Fig. 3*B*). Moreover, the threshold for evoking contralateral motor responses in both forelimb and hindlimb was lower than that for evoking ipsilateral motor responses (Wilcoxon signed rank test: *n* = 4 WT-Forelimbs, *p* = 0.12; 2 *Dcc*^+/−^-Forelimbs, *p* = 0.5; 11 WT-Hindlimbs, *p* = 9.7 × 10^−4^; 13 *Dcc*^+/−^-Hindlimbs *p* = 4.9 × 10^−4^; Fig. 3*C*), consistent with a proper contralateral projection of the corticospinal tract. In pairs of muscles recorded with EMGs, we found that the ipsilateral side was less excitable, sometimes not even reaching the threshold criterion in contrast to the contralateral side in both WT and *Dcc*^+/−^ mice (*n* = 6 WT and 6 *Dcc*^+/−^ mice; Fig. 3*D*). When there were sufficient ipsilateral EMG responses around the threshold, we calculated latencies on both the ipsilateral and contralateral side (Mann–Whitney test, *n* = 4 pairs, *p* = 0.74; Fig. 3*E*). Latencies of ipsilateral EMG responses occurred systematically after contralateral ones, but no differences were found between WT and *Dcc*^+/−^ mice. The strength of the response was evaluated as the number of motor spikes evoked on ICMS. Although the number of motor spikes in contralateral muscles appeared higher than in ipsilateral ones, it was not statistically different according to genotype (Wilcoxon signed rank test for contralateral vs ipsilateral responses: *n* = 6 WT, *p* = 0.22 and *n* = 6 *Dcc*^+/−^, *p* = 0.31. Mann–Whitney test for WT vs *Dcc*^+/−^: contralateral side, *p* = 0.31; ipsilateral side, *p* = 1; Fig. 3*F*). Furthermore, ICMS applied within the cortical representation of the hindlimb evoked specific motor responses in hindlimb muscles, but never in forelimb muscles, in the mutant mice (data not shown) and conversely the cortical representation of the forelimb never evoked any responses in hindlimb muscles, thus demonstrating that corticospinal projections maintain their specificity. Together, these results show that the projection and functional connectivity of the corticospinal tract are preserved in adult *Dcc*^+/−^ mice.

### Treadmill locomotion in the adult mouse

**Figure 4.**
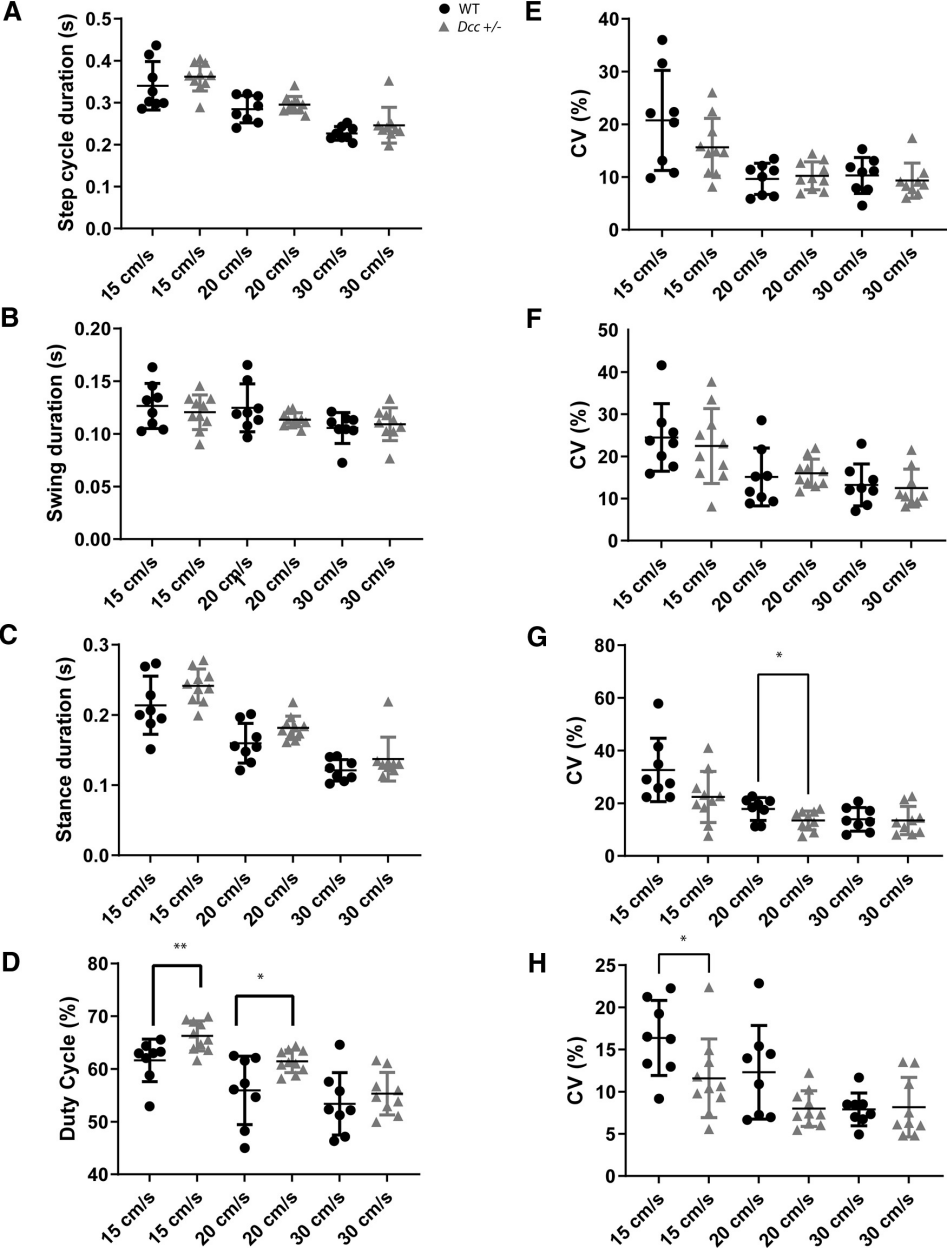
Locomotor pattern of adult *Dcc*^+/−^ and WT mice during treadmill locomotion. ***A–D***, Mean and (***E–H***) CV of step cycle duration (***A***, ***E***), swing duration (***B***, ***F***), stance duration (***C***, ***G***), and duty cycle of the stance phase (***D***, ***H***) of WT and *Dcc*^+/−^ mice at three different treadmill speeds (15, 20, and 30 cm/s). WT in black and *Dcc*^+/−^ in gray; *p < 0.05 and **p < 0.01 (for statistics, see Extended Data Table 4-1).

**Figure 5.**
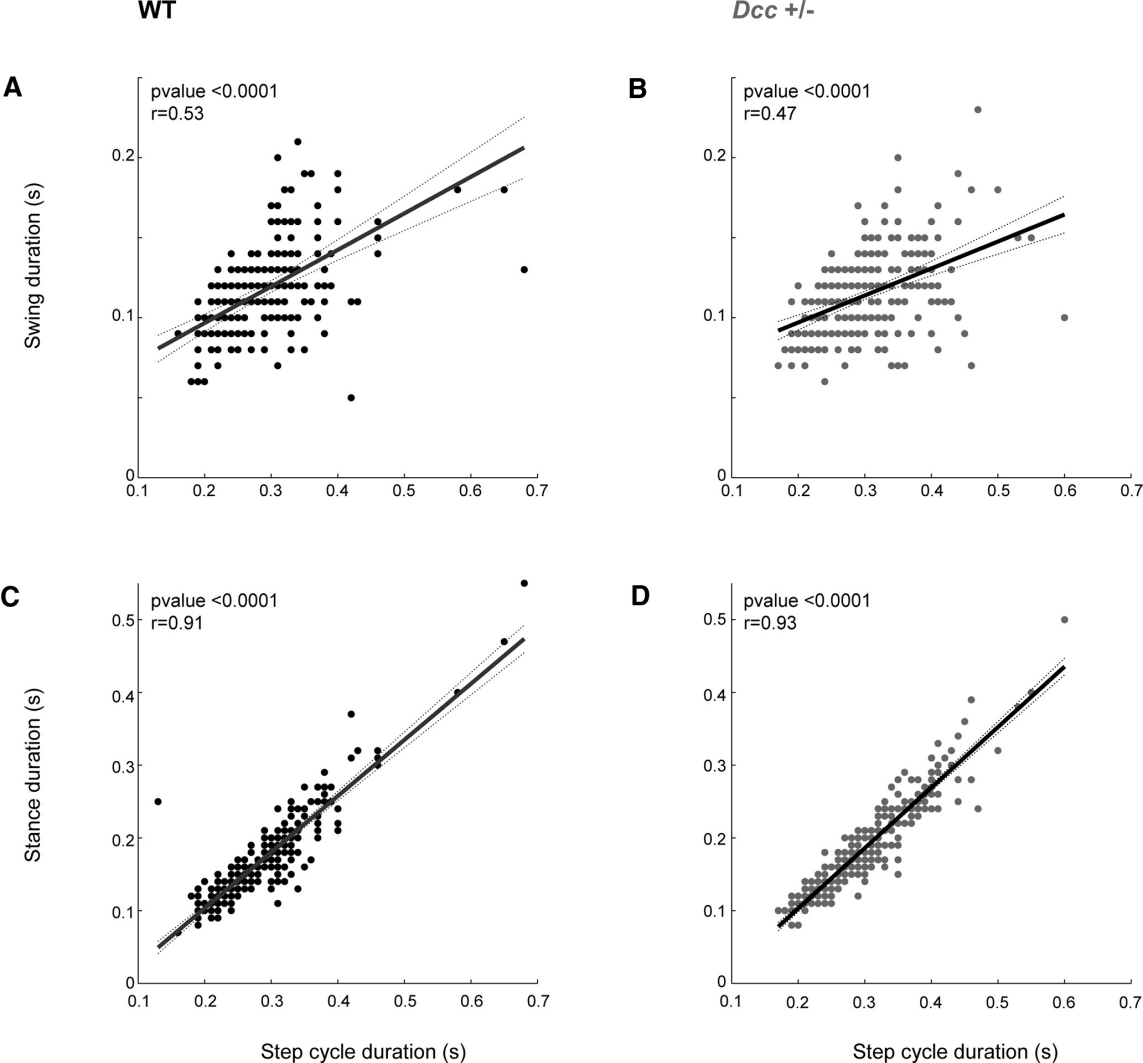
Swing and stance duration as functions of step cycle duration during treadmill locomotion. Swing (top panels, ***A***, ***B***) and stance (bottom panels, ***C***, ***D***) duration as functions of step cycle duration of WT and *Dcc*^+/−^ mice. Note three different treadmill speeds were combined. WT in black and *Dcc*^+/−^ in gray.

Although *Dcc*^+/−^ mice do not show impaired gross voluntary motor and locomotor behaviors, we hypothesized that the loss of one *Dcc* allele might cause more subtle changes in locomotor pattern and rhythm. To test that, we performed gait analysis of WT and *Dcc*^+/−^ mice during treadmill locomotion at steady speeds of 15, 20, and 30 cm/s. Overall, the mean and CV of the step cycle duration, the swing duration, the stance duration, and the duty cycle of the stance phase decreased as a function of treadmill speed for both WT and *Dcc*^+/−^ mice (Fig. 4). Although the duration of the step cycle and stance phase was normal, the duty cycle of the stance phase was significantly increased in *Dcc*^+/−^ mice in comparison to their WT littermates at low and intermediate treadmill speeds (Fig. 4*D*, *n* = 8 WT and 10 *Dcc*^+/−^ mice, duty cycle, Mann–Whitney test, *p* = 0.0085 at 15 cm/s; unpaired Student’s *t* test, *p* = 0.0490 at 20 cm/s; for statistics, see Extended Data Table 4-1). Moreover, we also found a significant decrease in the variability of the duty cycle of the stance phase in *Dcc*^+/−^ mice in comparison to WTs at 15 cm/s (Fig. 4*H*, *n* = 8 WT and 10 *Dcc*^+/−^ mice, duty cycle, unpaired *t* test, *p* = 0.0420 at 15 cm/s; for statistics, see Extended Data Table 4-1). To look for changes in locomotor pattern as function of speed, we then plotted the duration of the stance and swing phase as function of step cycle duration. The linear regression did not show any significant differences according to genotype (*n* = 8 WT and 10 Dcc^+/−^ mice, *F* test on slopes, *p* = 0.0503, *F*_(1,497)_ = 3.85; Fig. 5), thus suggesting overall a normal locomotor pattern in the *Dcc* heterozygous mice.

10.1523/ENEURO.0216-18.2021.t4-1 Extended Data Table 4-1 Locomotor pattern of adult *Dcc*^+/−^ and WT mice during treadmill locomotion. Download Table 4-1, DOC file.

**Figure 6.**
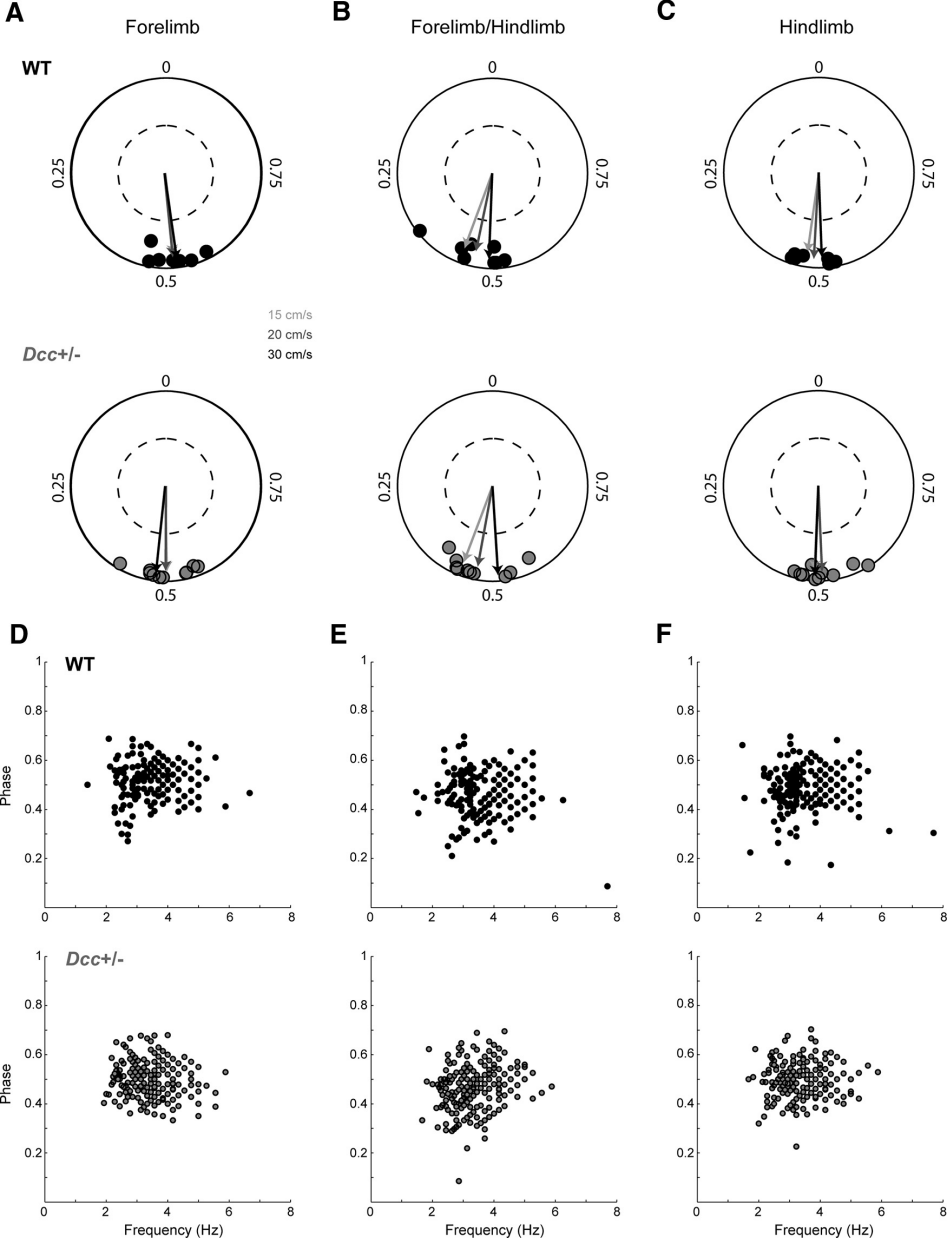
Bilateral and homolateral interlimb coordination during treadmill locomotion. ***A–C***, Polar plots showing the mean vector for the relationships between left and right forelimbs (***A***), left and right hindlimbs (***C***), and between homolateral forelimb and hindlimb (***B***) of WT (black circles) and *Dcc*^+/−^ (gray circles) at treadmill speeds of 15, 20, and 30 cm/s. The position on the polar plot indicates mean phase; the distance from the center of the polar plot indicates strength of the coupling (Rayleigh). Symbols represent individual mice at a treadmill speed of 20 cm/s, vectors represent the mean phase coupling of WT and *Dcc*^+/−^ groups at 15, 20, and 30 cm/s. Dashed inner circles represent a Rayleigh value of 0.5. ***D–F***, Phase of the coupling between left and right forelimbs (***D***), hindlimbs (***F***), and (***E***) forelimb-hindlimb as a function of locomotor frequency during treadmill locomotion at 15–30 cm/s. WT in black and *Dcc*^+/−^ in gray.

Given the bilateral locomotor disorganization in *Dcc* homozygous mutant spinal cords (Rabe Bernhardt et al., 2012), we also investigated the bilateral and homolateral coupling of limbs during treadmill locomotion. As shown by their polar plots (Fig. 6*A–C*), the coupling between left-right forelimbs and hindlimbs, as well as the homolateral coupling between forelimbs and hindlimbs, were normal in *Dcc*^+/−^ mice in comparison to their WT littermates (Watson–William test of WT vs *Dcc*^+/−^: hindlimb coupling, *p* = 0.71 at 15 cm/s, *p* = 0.70 at 20 cm/s and *p* = 0.99 at 30 cm/s; WT vs *Dcc*^+/−^: forelimb coupling, *p* = 0.77 at 15 cm/s, *p* = 0.99 at 20 cm/s and *p* = 0.71 at 30 cm/s; WT vs *Dcc*^+/−^: homolateral coupling, *p* = 0.98 at 15 cm/s, *p* = 0.69 at 20 cm/s and *p* = 0.95 at 30 cm/s). We also looked at phase coupling as function of locomotor frequency, which shows that the coordination between left and right forelimbs and hindlimbs was normal overall in *Dcc*^+/−^ mice during locomotion at treadmill speeds from 15 to 30 cm/s (Fig. 6*D–F*). Overall, these results show that locomotor pattern, rhythm, and interlimb coordination of *Dcc*^+/−^ adult mice are normal.

**Figure 7.**
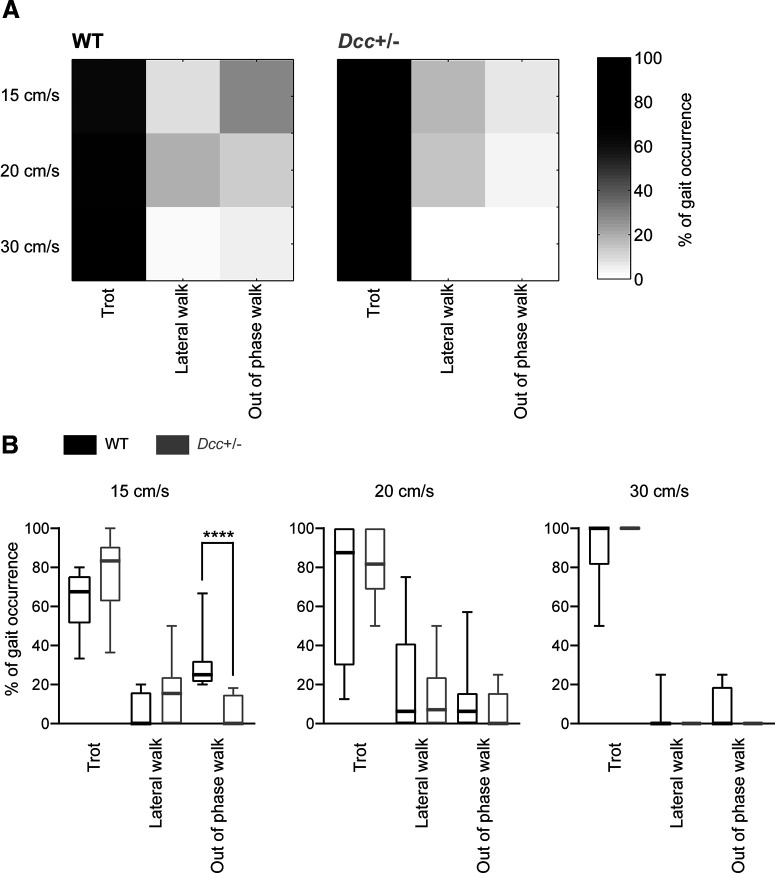
Locomotor gait occurrence during treadmill locomotion. ***A***, Gray-scaled matrixes of the percentage of occurrence of a gait (column) at 15, 20, and 30 cm/s (row) for WT and *Dcc*^+/−^ mice. The sum of a row equals 100%. ***B***, Box plots representing the percentage of gait occurrence at 15, 20, and 30 cm/s; ****p < 0.0001.

As their WT littermates, *Dcc*^+/−^ mice exhibited out-of-phase walk (i.e., asymmetrical walk), lateral walk, and trot with the predominance of trot at the highest treadmill speed tested of 30 cm/s (Fig. 7). Interestingly, *Dcc*^+/−^ mice exhibited significantly less out-of-phase walk than their WT littermates at the slowest treadmill speed of 15 cm/s (*n* = 8 WT and 10 *Dcc*^+/−^, Mann–Whitney test, *p* < 0.0001 at 15 cm/s; Fig. 7*B*); nevertheless, its occurrence normalized at higher treadmill speeds of 20 and 30 cm/s (no significant differences between *Dcc*^+/−^ and WT mice; Fig. 7*B*). Taken together, these analyses suggest that the repertoire of gaits was overall normal in *Dcc*^+/−^ mice.

### Locomotor pattern and rhythm during neonatal locomotor-like activity

**Figure 8.**
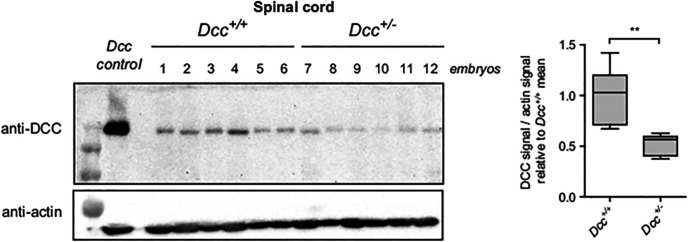
Dcc protein levels in *Dcc*^+/−^ and WT spinal cords. Left, Western blotting of Dcc in embryonic WT and *Dcc*^+/−^ spinal cords. *Dcc control* is a cell lysate overexpressing a *Dcc* cDNA. Right, Dcc/actin ratio relative to WT (*Dcc^+/+^*); **p < 0.01.

Given that motor and locomotor controls appear to be normal in adult *Dcc*^+/−^ mice, we then assessed whether the loss of one WT allele of *Dcc* could impair the function of isolated local spinal locomotor circuits. We first verified whether *Dcc*^+/−^ developing spinal cords have reduced Dcc protein levels. Western blottings showed that Dcc protein levels are reduced by ∼50% in *Dcc*^+/−^ embryonic spinal cords compared with WT spinal cords (n = 6 embryos per genotype, Mann–Whitney test, *p* = 0.002; Fig. *8*). This is consistent with what has been observed previously in adult *Dcc*^+/−^ mouse spinal cords (Liang et al., 2014).

**Figure 9.**
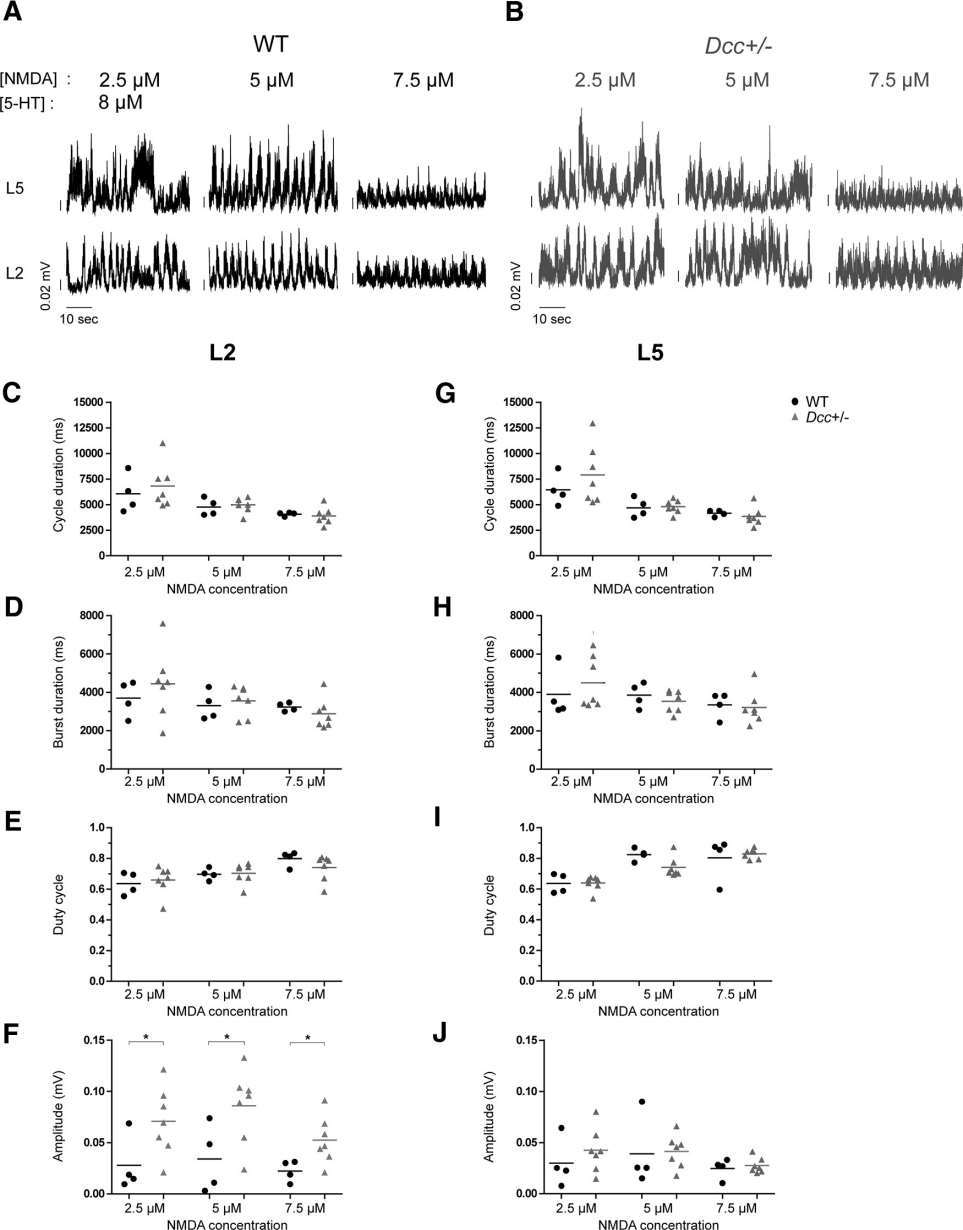
Locomotor pattern and rhythm during neonatal locomotor-like activity. ***A***, ***B***, Examples of L5 and L2 ENG recordings of WT (***A***) and *Dcc*^+/−^ (***B***) mice on bath application of drugs (8 μm 5HT and 2.5, 5, or 7.5 μm NMDA). ***C–J***, Mean and data of cycle duration (***C***, ***G***), burst duration (***D***, ***H***), duty cycle (***E***, ***I***), and burst amplitude (***F***, ***J***) of WT and *Dcc*^+/−^ L2 (***C–F***) and L5 (***G–J***) ENGs at different NMDA concentrations (8 μm 5HT and 2.5, 5, or 7.5 μm NMDA); *p < 0.05 (for statistics, see Extended Data Table 9-1).

We next assessed whether the loss of one WT allele of *Dcc* could impair the function of isolated local spinal locomotor circuits. To test this, we used spinal cords isolated from neonatal WT and *Dcc*^+/−^ mice, thus allowing us to study spinal circuits without the influence of descending or peripheral input. As previously described (Kudo and Yamada, 1987; Cazalets et al., 1992), locomotion was triggered by bath application of a cocktail of 8 μm 5HT and 2.5, 5, or 7.5 μm NMDA to challenge spinal interneuronal excitability. As illustrated by ENG bursts of L2 and L5 lumbar ventral root activities (Fig. 9*A*,*B*), locomotor-like activity was evoked at a low concentration of 2.5 μm NMDA, but the activity was more regular and stable at an intermediate concentration of 5 μm before decreasing in amplitude at a high concentration of 7.5 μm in both WT and *Dcc*^+/−^ mutant spinal cords (Fig. 9*F*). Increasing the concentration of NMDA statistically decreased the cycle duration, which translated into an increased duty cycle in both L2 and L5 ENG bursts for both WT and *Dcc*^+/−^ spinal cords (Fig. 9*D*). Nevertheless, no significant changes were found in cycle duration, burst duration, or duty cycle regarding the mouse genotype (for statistics, see Extended Data Table 9-1), thus suggesting normal functioning of the spinal interneuronal circuit in *Dcc*^+/−^ mice.

10.1523/ENEURO.0216-18.2021.t9-1 Extended Data Table 9-1 Locomotor pattern and rhythm during neonatal locomotor-like activity. Download Table 9-1, DOC file.

As shown in both WT and *Dcc*^+/−^ spinal cord examples (Fig. 9*A*,*B*), the ENG burst amplitude in L2 and L5 increased as a function of NMDA concentration from 2.5 to 5 μm and tended to decrease at a high concentration of 7.5 μm, below the amplitude level evoked at 2.5 μm. Interestingly, the amplitude of the L2 ENG burst of *Dcc*^+/−^ spinal cords was significantly higher than that of WTs regardless of NMDA concentration (Fig. 9*F*, *n* = 4 WT and 7 *Dcc*^+/−^, burst amplitude of WT vs *Dcc*^+/−^ L2 ENGs, Mann–Whitney test, *p* = 0.0424 at 2.5 μm, *p* = 0.0424 at 5 μm, *p* = 0.0242 at 7.5 μm; for statistics, see Extended Data Table 9-1). This increased motor output suggests that the spinal locomotor circuit (at least in the L2 segment) is more excitable on bath application of NMDA in the developing spinal cord on loss of one *Dcc* allele.

### Variability in locomotor pattern and rhythm during neonatal locomotor-like activity

**Figure 10.**
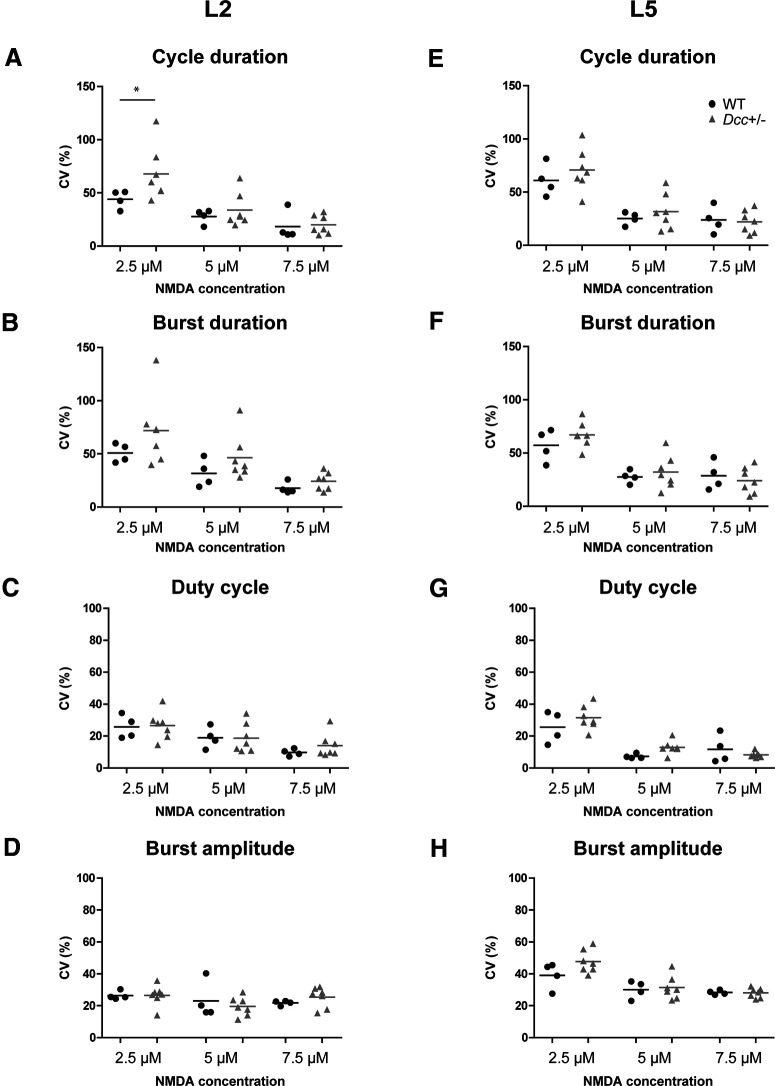
Variability in ENG waveforms during neonatal locomotor-like activity. Mean and CVs of cycle duration (***A***, ***E***), burst duration (***B***, ***F***), duty cycle (***C***, ***G***), and burst amplitude (***D***, ***H***) of WT (black circles) and *Dcc*^+/−^ (gray triangles) of L2 (***A–D***) and L5 (***E–H***) ENG waveforms at different NMDA concentrations; *p < 0.05 (for statistics, see Extended Data Table 10-1).

As previously shown in some mutant mouse studies (Zhang et al., 2008), *Dcc*^+/−^ mutation might translate into a higher variability in locomotor pattern and rhythm during locomotion. To test this hypothesis, we quantified the CV in locomotor features (Fig. 10). Although ENG waveforms were more variable at low concentrations of NMDA (Fig. 9*A*,*B*), overall, there were no significant differences in the variability of cycle duration, burst duration, burst amplitude, and duty cycle (Fig. 10*A*, *n* = 4 WT and 7 *Dcc*^+/−^, Mann–Whitney test for L2 cycle duration, *p* = 0.0242; for statistics, see Extended Data Table 10-1). Only the cycle duration of the L2 ENG waveform showed significantly higher variability in the *Dcc*^+/−^ cycle duration of the L2 ENG in comparison to the WT.

10.1523/ENEURO.0216-18.2021.t10-1 Extended Data Table 10-1 Variability in ENG waveforms during neonatal locomotor-like activity. Download Table 10-1, DOC file.

### Left-right and flexor-extensor coordination during neonatal locomotor-like activity

**Figure 11.**
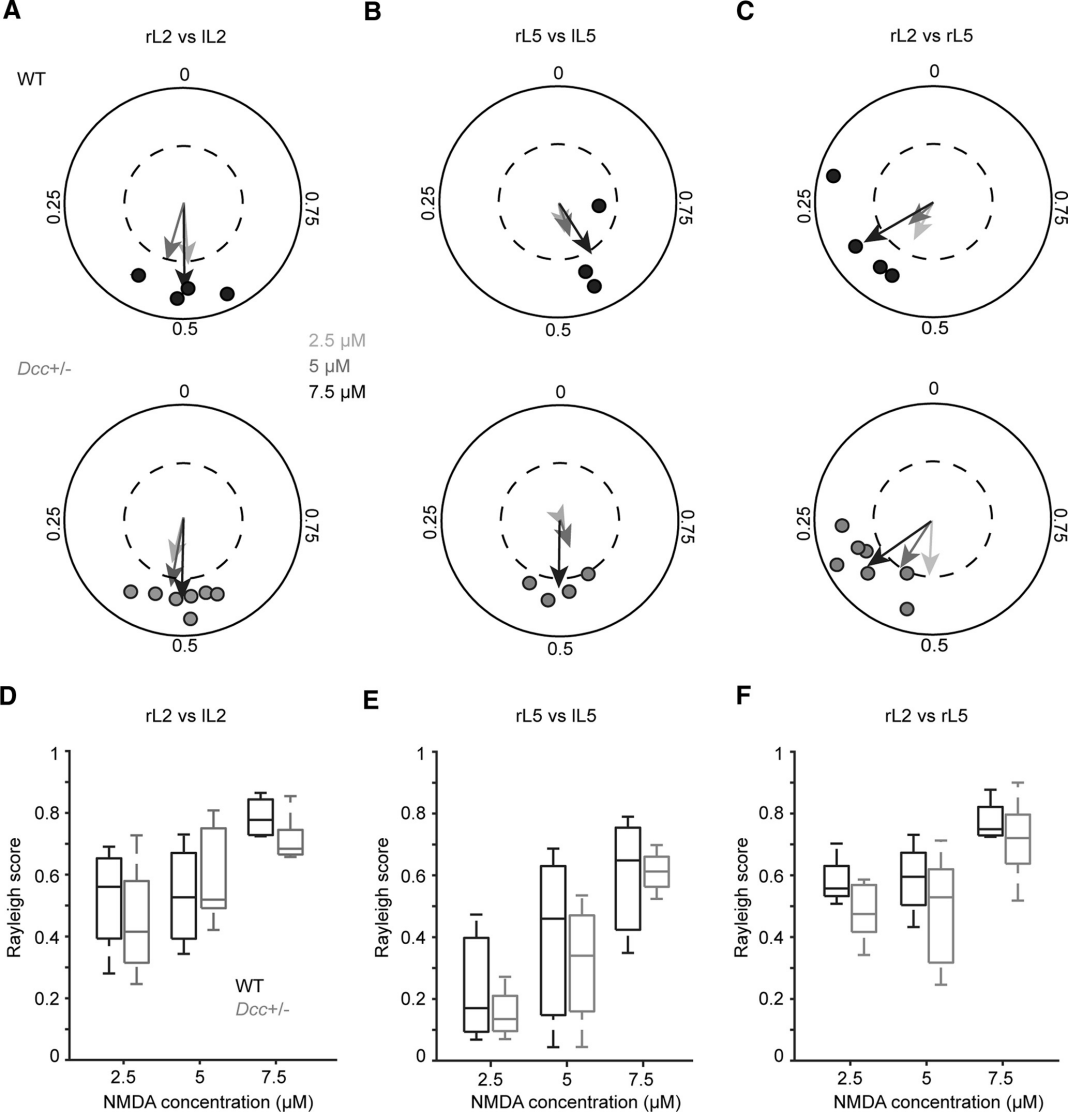
Locomotor coupling during neonatal locomotor-like activity. ***A–C***, Polar plots showing the mean vector (arrows) for the relationships between left and right L2 (***A***, lL2 vs rL2) and L5 (***B***, lL5 vs rL5), and between homolateral flexor and extensor (***C***, rL2 vs rL5) of WT (top polar plots) and *Dcc*^+/−^ (bottom polar plots) spinal cord preparations at low (2.5 μm, lighter gray arrow), intermediate (5 μm, darker gray arrow), and high (7.5 μm, black arrow) NMDA concentrations. The position on the polar plot indicates mean phase; the distance from the center of the polar plot indicates strength of the coupling (Rayleigh). For clarity, individual data are shown only for the highest concentration (black symbols for WT and gray symbols for *Dcc*^+/−^). Dashed inner circles represent a Rayleigh value of 0.5. ***D–F***, Boxplots of the Rayleigh score at three NMDA concentrations between left and right L2 (***D***), left and right L5 (***E***), and between homolateral L2 and L5 (***F***). rL2 = right L2 ventral root; lL2 = left L2 ventral root; rL5 = right L5 ventral root; lL5 = left L5 ventral root (for statistics, see Extended Data Table 11-1).

Although some mutant mice can produce a more variable locomotor coordination (Zhang et al., 2008), others produce a less variable one (Bellardita and Kiehn, 2015). As illustrated by polar plots (Fig. 11; for statistics, see Extended Data Table 11-1), the coordination between left-right flexor (Fig. 11*A*), left-right extensor (Fig. 11*B*), and flexor-extensor (Fig. 11*C*) related ventral root activities were not significantly different according to the genotype (Watson–William test of WT vs *Dcc*^+/−^: left-right L2 coupling, *p* = 0.742 at 2.5 μm, *p* = 0.890 at 5 μm and *p* = 0.973 at 7.5 μm; WT vs *Dcc*^+/−^: left-right L5 coupling, *p* = 0.880 at 2.5 μm, *p* = 0.954 at 5 μm and *p* = 0.582 at 7.5 μm; WT vs *Dcc*^+/−^: right L2-L5 coupling, *p* = 0.705 at 2.5 μm, *p* = 0.840 at 5 μm and *p* = 0.894 at 7.5 μm). The variability of the coupling was not affected in *Dcc*^+/−^ mice as evaluated with Mann–Whitney tests on Rayleigh values of WT versus *Dcc*^+/−^ left-right L2 coupling: *p* = 0.527 at 2.5 μm, *p* = 0.527 at 5 μm, and 0.109 at 7.5 μm (Fig. 11*D*); WT versus *Dcc*^+/−^ left-right L5 coupling: *p* = 0.629 at 2.5 μm, *p* = 0.629 at 5 μm, and 0.857 at 7.5 μm (Fig. 11*E*); WT versus *Dcc*^+/−^ rL2-rL5 coupling, *p* = 0.230 at 2.5 μm, *p* = 0.412 at 5 μm, and 0.648 at 7.5 μm (Fig. 11*F*). Overall, these results suggest a normal flexor-extensor and left-right coordination regardless of the NMDA concentrations in *Dcc*^+/−^ spinal cord preparations.

10.1523/ENEURO.0216-18.2021.t11-1 Extended Data Table 11-1 Locomotor coupling during neonatal locomotor-like activity. Download Table 11-1, DOC file.

## Discussion

We show that adult mice lacking one allele of functional *Dcc* do not show impairments in skilled motor and locomotor control. In contrast to human individuals who have heterozygous *DCC* mutations, *Dcc* heterozygosity in mice does not promote an aberrant bilateral projection of the corticospinal tract or prevents its normal contralateral projection. The integrity of cortical representations and the functional connectivity of the corticospinal tract to forelimb and hindlimb motoneuronal pools are also preserved in *Dcc*^+/−^ mice. On the other hand, although treadmill locomotion is mostly normal, the loss of one *Dcc* allele increases the duration and duty cycle of the stance phase, suggesting sensory feedback impairment. Moreover, although the spinal locomotor circuit appears functionally normal, *Dcc* heterozygous mutation increases the output of the flexor-related motoneuronal pool of isolated neonatal spinal cords. In summary, the heterozygous *Dcc* mouse does not replicate the motor phenotype of people affected with *DCC* haploinsufficiency, but it exhibits subtle motor differences that have not been reported so far.

### Skilled motor control, motor cortex, and its corticospinal tract in adult *Dcc*^+/−^ mice

Although the corticospinal tract and connectivity in the mouse are different from that of the human, previous studies using a spontaneous mutation allele that removes the exon encoding the P3 intracellular domain of DCC have shown that homozygous *Dcc*^kanga/kanga^ and *Dcc*^kanga/^*^−^* mice exhibit a hopping gait, ataxia, and abnormal pyramidal decussation through adulthood, thus recapitulating to some extent the motor phenotype of *DCC* haploinsufficient individuals (Finger et al., 2002; Welniarz et al., 2017). We therefore examined *Dcc* heterozygous mice and assessed the contribution of *Dcc* to skilled motor and locomotor control, which relies on the integrity of the motor cortex and its corticospinal tract (Pourchet et al., 2021). We found that *Dcc*^+/−^ mice exhibited normal asymmetrical control of the forelimb during vertical exploration in the cylinder test; they also exhibited normal skilled forelimb coordination during voluntary locomotor control while walking on a wide and narrow beam and while crossing a horizontal ladder. Moreover, ICMS applied within cortical representations of the forelimb versus hindlimb evoked specific motor responses in forelimb or hindlimb muscles, respectively, in the *Dcc*^+/−^ mice, thus supporting the hypothesis that cortical areas and their corticospinal projections maintain their specificity. The absence of differences in cortical representations and the absence of differences in synaptic connectivity or latency of the corticospinal tract axons to the spinal cord in *Dcc*^+/−^ mice argue that cortical representation and functional connectivity of the corticospinal tract are normal in *Dcc*^+/−^ mice.

### Increased duration of the duty cycle of the stance phase of adult *Dcc*^+/−^ mice during treadmill locomotion

In contrast to the hopping gait of *Dcc^kanga/kanga^* and *Dcc^kanga/−^* mutant mice (Finger et al., 2002; Welniarz et al., 2017) or conditional ablation of *Dcc* in *HoxB8*-expressing spinal neurons (Peng et al., 2018), we found no defects in locomotor gait of adult *Dcc*^+/−^ mice during treadmill locomotion. Although we only assessed *Dcc*^+/−^ mice at slow and intermediate walking speeds, no events of hop or gallop were observed during brief locomotor accelerations when the animals sped up to reach a locomotor frequency of 5–6 Hz. Among our locomotor data, the duty cycle of the stance phase was significantly increased in *Dcc*^+/−^ mice, especially at slow walking speed. Given the absence of changes in the duration of flexor and extensor-related locomotor activities and in their coupling during locomotor-like activity even at high NMDA concentrations using isolated spinal cord preparations, the increased duration of the duty cycle of the stance phase of *Dcc*^+/−^ mice might reflect a sensory feedback deficit. In support to this idea, Netrin-1/Dcc signaling guides sensory axons (Lakhina et al., 2012; Laumonnerie et al., 2014): *Netrin-1* and *Dcc*^−/−^ spinal cords exhibit an aberrant projection of cutaneous and proprioceptive axons in the spinal cord (Watanabe et al., 2006; Masuda et al., 2008; Laumonnerie et al., 2014) and *Dcc* is required for the normal development of nociceptive processing in mice and humans (da Silva et al., 2018). Furthermore, removing sensory afferents of semi-intact spinal cord preparations shortens the duration of the extensor phase during locomotion (Juvin et al., 2007); therefore, an aberrant sensory feedback in *Dcc*^−/−^ mice could presumably increase the duty cycle of their stance phase. Further studies will be necessary to test this hypothesis in mice and humans with the heterozygous mutation.

### Spinal locomotor circuits are normal but show increased motoneuronal output modulation in neonatal *Dcc*^+/−^ mice

Using spinal cords isolated from neonatal mice, we also investigated the spinal locomotor circuit in the absence of descending input from the brain and sensory feedback from the periphery. In contrast to *Dcc*^−/−^ or *Dcc*^kanga/kanga^ spinal cords (Rabe Bernhardt et al., 2012), those with *Dcc*^+/−^ mutation had normal coordination between left and right and between flexor-related and extensor-related motoneuronal output modulation during neonatal locomotion. Moreover, there were no significant differences in locomotor pattern and rhythm regardless of NMDA concentrations. However, the amplitude of the flexor-related motoneuronal activity was significantly increased in *Dcc*^+/−^ spinal cords on bath application of NMDA, suggesting that DCC plays a role in the establishment of the neuronal circuit modulating the excitability of the spinal locomotor circuit. Nevertheless, this increased output in flexor-related motoneuronal activity did not persist through adulthood, suggesting that sensory feedback might readjust the motoneuronal output of adult mutant mice. Perhaps comparative EMG studies of stepping in newborn, adolescent, and adult humans carrying monoallelic *DCC* mutations would reveal similar abnormalities during bipedal walking or quadrupedal crawling (Patrick et al., 2009; Vasudevan et al., 2016).

In summary, our study finds that, in contrast to humans, a heterozygous mutation in *Dcc* has little effect on skilled and basic locomotor control, or on the normal functioning of the motor cortex and its corticospinal connectivity. Although no functional impairments were found either in locomotor pattern and rhythm of spinal cord preparations isolated from neonatal mice, the flexor-related motoneuronal output was significantly increased in the developing spinal cord of *Dcc*^+/−^ mice. However, this increase in flexor-related motoneural output did not persist through adulthood and locomotor gait was overall normal, albeit with a longer duty cycle and less out-of-phase walk. This finding raises the possibility of the existence of subtle locomotor changes in humans carrying monoallelic *DCC* mutations.

## Synthesis

Reviewing Editor: Silvia Pagliardini, University of Alberta Decisions are customarily a result of the Reviewing Editor and the peer reviewers coming together and discussing their recommendations until a consensus is reached. When revisions are invited, a fact-based synthesis statement explaining their decision and outlining what is needed to prepare a revision will be listed below. The following reviewer(s) agreed to reveal their identity: Adolfo Talpalar.

REVIEW SUMMARY

This manuscript is a resubmission of a previous version which was rejected. The study focuses on the motor consequences of partial DCC mutations in mice, which are produced in heterozygote DCC+/- mice. Human monoallelic DCC mutations lead to mirror movements. Although there have been several studies which have detailed other DCC mutations in mouse, this is the first to fully describe the heterozygous DCC+/- mouse.

The authors demonstrate that there is no overt motor phenotype in adults or in neonatal cords. However, there are a few subtle differences in the heterozygous DCC mice including an increase stance phase in adult treadmill locomotion and an increase in the amplitude of flexor motor output during in vitro locomotor-like activity in the neonatal isolated cord. They conclude that humans with monoallelic DCC mutations may also express similar subtle locomotor changes, in addition to the more obvious mirror movements.

The rationale for studying the heterozygous/monoallelic mutant is well reasoned based on the human mutation but expectations of mirror movements in heterozygous rodents are less so based on mouse work using variations of DCC mutants. Evidence for differences in CST projections is lacking from the prior mouse work (Welniarz et al. 2017), as mentioned. Similarly, major effects in locomotor-like activity in vitro would not be expected because DCC+/- mice were combined with controls for electrophysiology (Rabe Berhardt et al 2012) and spinal commissural projections in DCC+/- (Fazeli et al 1997) and Hoxb8cre; DCC+/fl (Peng et al 2017) mice were similar to controls and used as controls, respectively. Nonetheless, there were more subtle phenotypes found that were not detected by prior studies. The study is thorough, rigorous, well designed, well-executed, and largely falls in line with the findings from the prior studies. The data, although mainly negative, are convincing. The apparent additions to this revised manuscript, including Western blots, behavioral tests, and the ICMS benefit the study and allow for more solid conclusions to be drawn. The manuscript is very well written and logical, and the figures are clear (with some exceptions noted below).

Major comments:

A more careful appreciation of the results could enhance the significance of the study. The results in fact show that the same genetic mutation that causes motor defect in humans does not provide similar outcome in mice.

Comparing human physiopathology with animal models displaying similar genetic changes is important and recurrent. However, the results are interesting not only if the model mimics human behavior, but also if it DIVERT from human. With some minor exceptions, the current manuscript displays an interesting collection of WELL-DESCRIBED NEGATIVE data. These findings could provide authors an opportunity to discuss the following: Should transgenic mice model human behavior because of suffering ’similar’ genetic changes? Should a mouse model mimic all human physiology (and physiopathology)? Even if there are some resemblances, I don’t believe that animals can model ALL human physiology (animal physiology is interesting enough not to resemble us) as we can’t model other animals. The authors’ data, if properly interpreted, introduced and discussed, can make a great paper that mice ARE NOT a universal model of human physiology (even when knocking out the same gene). I envisage a comment on it in eNeuro saying ’Mice are not human: lessons from motor function’. A pretty obvious situation, which is being distorted for quite a while.

For such goal the manuscript should be organized to display properly known features to show what is obvious from the results: a) that human motor networks and physiology are DIFFERENT from rodents, and b) that a similar genetic change produces a different outcome.

Introduction is a bit disorganized and facts should be dissociated from hypotheses. The cortical part of the introduction is extremely long. It should be shortened. If the authors want to refer to hopping (as a kind of mirror-movement) they can’t disregard robust evidence that left-right alternation in rodents is normally secured by commissural SPINAL V0 neurons (Lanuza et al., 2004; Talpalar et al., 2013; Bellardita & Kiehn, 2015; Zelenin et al., 2021), while hopping is produced by unbalance between synchronizing and alternating influences in the spinal cord either by overexpression of excitatory axons to the other side (Kullander et al, 2003) or by elimination of the normal V0 commissural interneurons projecting to the contralateral side during locomotion and in vitro locomotor-like activity in the lumbar region, and not by any supraspinal tract (Talpalar et al., 2013). A recent study shows that such unbalance does not exclusively affect locomotion but also other rhythmic and non-rhythmic motor activities (Zelenin et al., 2021).

Pages 3-4 Lines 51-54 The authors attribute hopping in the rodent model to cortical or corticospinal influences (CST). This is not correct. Even if there is alteration of corticospinal tracts in DCCkanga-kanga, I dispute that they produce hopping. I rather postulate that it’s a coincidence. Hopping occurs almost exclusively when SPINAL commissural tracts are affected (Talpalar et al., 2013). All these studies show that hopping occurs either when commissural inhibition is eliminated (Zelenin et al., Talpalar et al) or when originally ipsilateral excitatory fibers anomalously cross the midline (Kullander et al., 2003) ALWAYS at a spinal level. To my knowledge, nobody has ever shown hopping occurring because of supraspinal tract alteration in rodents. Moreover, unlike humans’ the CST of rodents does not directly project to motorneuron pools. Please, correct the Introduction according to these facts.

Results section should be adapted to fit the general line of the manuscript: that heterozygote DCC suppression produce mirror movement in humans but not in mice.

Lines 223- 227 I don’t know what is ’voluntary’ in mice. I guess that the authors should include forelimb reaching as the closest estimate of ’voluntary movements’ in mice. But forelimb show preserved normal asymmetry in the experiments.

At most treadmill speeds, there is a mix of gaits expressed which may influence the data. Alternating gaits will have similar results in phasing plots but there are clear differences when examining the step order and duration - which are less obvious than full alternation vs full synchrony and may be expected with a weaker phenotype. Were the gaits (i.e. trot, lateral walk, out of phase walk - see Lemieux et al 2016) expressed in similar percentages by DCC heterozygous mice and wildtype mice?

All locomotion (in vivo and in vitro) is relatively slow/low frequency locomotion. V0 and V3 neurons should be the ones affected with the DCC mutation and these have been shown to be active in a speed-dependent manner. Could there be locomotor phenotypes at other speeds? The locomotor effects of the removal of the V0V population were not seen until frequencies greater than 0.35-0.4 Hz (Talpalar et al 2013, which corresponds to 2500-2850ms in Fig 8). Such frequencies should be possible with >7.5uM NMDA. Minimally, this should be mentioned in the discussion.

Burst amplitudes are highly dependent on the seal of the suction electrode (size of the root, size of the electrode, etc). Given that this is one of the main positive findings of the study, some detail should be added to the Methods about the electrodes (were they all of a standard size or selected based on size relative to root?) and about the measurement of amplitude (if from an integrated signal, was amplitude measured from the baseline (0) or the background activity/noise).

The role of cortex in the control of locomotion in rodents is not clear. Therefore, the authors’ assumption that cortex produces ’voluntary locomotor control’ is a bit too anthropocentric. It is known that mice, and even cats, can produce reasonable locomotion without cortex (see classic literature like Grillner’s review 1981; Skik and Orlovsky, 1966, etc.). There is no need for such a dubious claim. The authors study spontaneous motor control and locomotion.

Lines 324-325 Can treadmill locomotion be considered ’voluntary’? The treadmill evokes or initiate locomotion but locomotor control is dominantly under spinal and maybe some subcortical structure control. Mice may ’willingly’ engage in following the treadmill or not, but the locomotion performance is nearly automatic. I don’t think that mice are able to ’voluntarily’ change their gaits like humans can eventually do. Please, eliminate ’voluntary’ where not properly applied.

Physiological assessment of CST by electrical stimulation. Mice data is COMPLETELY different from human. Electric or TMS stimulation in humans has a short delay. Humans CST conveys directly to motor pools, while rodent CST is projected to the dorsal horn. So, pathways are different. Human motor control is highly dependent of cortex while this is not the case in other animals like cats and mice. The fact cortical stimulation produces some movement in mice hind limbs has a more complex explanation than in humans. Please, adapt the text considering these known differences. Moreover, the fact that the authors need a train of stimuli to evoke CST induction of burst on TA muscle adds complexity to the interpretation of a signal that occurs about >40 ms after the first stimulus. Therefore the physiological interpretation of this long latency measurement is not straightforward to me. Though, it’s clear that is not changed in DCC+/- animals.

Figure 10 Some mutants produce more variance like the cited studies(Zhang et al, 2008), but other produce LESS variance (Bellardita and Kiehn, 2015). The authors should cite more widespread the highly relevant literature...

The differences that the authors see during treadmill locomotion and during in vitro locomotor-like recordings are MINIMAL. I don’t think that they reflect any highly specific change. The number of animals is not large. I wouldn’t draw tremendous conclusion from such a small sample size.

Figure 11. There is no proper rationale for the use of burst deletions in this work. Which kind of information it conveys? What does it mean? The results are minimal if any and at the lowest NMDA concentration (see Talpalar and Kiehn, 2010 for proper methods and rationale if the authors want). I propose to eliminate those data from the manuscript. As such it doesn’t provide any useful information.

In the discussion, the authors insist on hypothesizing that the minor changes observed in the gait in their mouse model explain altered movement in human DCC heterozygotes. I think that the small changes that the authors see have nothing to do with mirror movements or with human pathophysiology. I infer that mirror movement occur in humans because their cortical motor function is dominant compared with rodents which rather rely on subcortical and spinal networks for motor function. Please, emphasize this difference. It’s everywhere in classic motor literature.

The changes that the authors see in the gait and locomotor-like activity are MINOR and the number of animals is small. I don’t think that it makes any sense trying to predict that they have anything to do with the robust features of human pathology in adults. Do DCC deficient humans show mirror movements since birth? I don’t know. If they don’t, then mirror movements are not innate, and may be learnt while learning motor and locomotor functions later in life. Please, shorten the discussion and focus it on the fact that mice DO NOT mimic human mirror movements.

Minor issues

Page 3 Line 53 I don’t think that description of hopping in a tetrapod can be ’kangaroo-like’ provided that kangaroos have a special bipedal or tripod gait with contribution of the tail. I think that a tetrapod instance in more appropriate, rabbit-like for example.

References: The layout of the different references is inconsistent. References layout should follow the journal guidelines for references.

The conclusion in lines 381-384 (’more resilient to high NMDA concentrations ....upon loss of one Dcc allele’.) is strong given that there is no statistically significant change with concentration of drug in either wt or DCC+/-.

Figure 6: Color coding in A-C is difficult to differentiate between 15 and 20cm/sec, given the line width of the vectors. The description in the legend (i.e. “WT(black circles)”) does not match what is shown. Are the points in A-C just at one speed but vectors at 3? The “dashed inner circles” are not dashed.

Since differences in left-right coordination deficits are those most consistent with the DCC-/- mutant phenotype and the lack of deficit fits with the Rabe Bernhart study, I suggest putting the phasing (Fig 10) ahead of or integrated with Fig 8, prior to going to the subtleties.

Lines 518-522 are not clear. They should be split into two sentences and collapsed MNs should be explained.

Lines 522-524 - Abnormal excitability in MNs could also be abnormal drive to them.

## Author Response

Responses to reviewers

29th Nov 2021

Synthesis Statement for Author (Required):

REVIEW SUMMARY

This manuscript is a resubmission of a previous version which was rejected. The study focuses on the motor consequences of partial DCC mutations in mice, which are produced in heterozygote DCC+/- mice. Human monoallelic DCC mutations lead to mirror movements. Although there have been several studies which have detailed other DCC mutations in mouse, this is the first to fully describe the heterozygous DCC+/- mouse.

The authors demonstrate that there is no overt motor phenotype in adults or in neonatal cords. However, there are a few subtle differences in the heterozygous DCC mice including an increase stance phase in adult treadmill locomotion and an increase in the amplitude of flexor motor output during in vitro locomotor-like activity in the neonatal isolated cord. They conclude that humans with monoallelic DCC mutations may also express similar subtle locomotor changes, in addition to the more obvious mirror movements.

The rationale for studying the heterozygous/monoallelic mutant is well reasoned based on the human mutation but expectations of mirror movements in heterozygous rodents are less so based on mouse work using variations of DCC mutants. Evidence for differences in CST projections is lacking from the prior mouse work (Welniarz et al. 2017), as mentioned. Similarly, major effects in locomotor-like activity in vitro would not be expected because DCC+/- mice were combined with controls for electrophysiology (Rabe Berhardt et al 2012) and spinal commissural projections in DCC+/- (Fazeli et al 1997) and Hoxb8cre; DCC+/fl (Peng et al 2017) mice were similar to controls and used as controls, respectively. Nonetheless, there were more subtle phenotypes found that were not detected by prior studies. The study is thorough, rigorous, well designed, well-executed, and largely falls in line with the findings from the prior studies. The data, although mainly negative, are convincing. The apparent additions to this revised manuscript, including Western blots, behavioral tests, and the ICMS benefit the study and allow for more solid conclusions to be drawn. The manuscript is very well written and logical, and the figures are clear (with some exceptions noted below).

We thank the reviewers for noting that our study is “thorough, rigorous, well designed, well-executed” and that “The data, although mainly negative, are convincing.” We are happy that the reviewer noted that the “additions to this revised manuscript, including Western blots, behavioral tests, and the ICMS benefit the study and allow for more solid conclusions to be drawn.”

We agree with the reviewer that “Although there have been several studies which have detailed other DCC mutations in mouse, this is the first to fully describe the heterozygous DCC+/- mouse.”

Finally, we also thank the reviewers for stating that “The manuscript is very well written and logical, and the figures are clear (with some exceptions noted below).” Below, we address the reviewers’ comments.

Major comments:

A more careful appreciation of the results could enhance the significance of the study. The results in fact show that the same genetic mutation that causes motor defect in humans does not provide similar outcome in mice.

Comparing human physiopathology with animal models displaying similar genetic changes is important and recurrent. However, the results are interesting not only if the model mimics human behavior, but also if it DIVERT from human. With some minor exceptions, the current manuscript displays an interesting collection of WELL-DESCRIBED NEGATIVE data. These findings could provide authors an opportunity to discuss the following: Should transgenic mice model human behavior because of suffering ’similar’ genetic changes? Should a mouse model mimic all human physiology (and physiopathology)? Even if there are some resemblances, I don’t believe that animals can model ALL human physiology (animal physiology is interesting enough not to resemble us) as we can’t model other animals. The authors’ data, if properly interpreted, introduced and discussed, can make a great paper that mice ARE NOT a universal model of human physiology (even when knocking out the same gene). I envisage a comment on it in eNeuro saying ’Mice are not human: lessons from motor function’. A pretty obvious situation, which is being distorted for quite a while.

For such goal the manuscript should be organized to display properly known features to show what is obvious from the results: a) that human motor networks and physiology are DIFFERENT from rodents, and b) that a similar genetic change produces a different outcome.

Introduction is a bit disorganized and facts should be dissociated from hypotheses. The cortical part of the introduction is extremely long. It should be shortened. If the authors want to refer to hopping (as a kind of mirror-movement) they can’t disregard robust evidence that left-right alternation in rodents is normally secured by commissural SPINAL V0 neurons (Lanuza et al., 2004; Talpalar et al., 2013; Bellardita & Kiehn, 2015; Zelenin et al., 2021), while hopping is produced by unbalance between synchronizing and alternating influences in the spinal cord either by overexpression of excitatory axons to the other side (Kullander et al, 2003) or by elimination of the normal V0 commissural interneurons projecting to the contralateral side during locomotion and in vitro locomotor-like activity in the lumbar region, and not by any supraspinal tract (Talpalar et al., 2013). A recent study shows that such unbalance does not exclusively affect locomotion but also other rhythmic and non-rhythmic motor activities (Zelenin et al., 2021).

Pages 3-4 Lines 51-54 The authors attribute hopping in the rodent model to cortical or corticospinal influences (CST). This is not correct. Even if there is alteration of corticospinal tracts in DCCkanga-kanga, I dispute that they produce hopping. I rather postulate that it’s a coincidence. Hopping occurs almost exclusively when SPINAL commissural tracts are affected (Talpalar et al., 2013). All these studies show that hopping occurs either when commissural inhibition is eliminated (Zelenin et al., Talpalar et al) or when originally ipsilateral excitatory fibers anomalously cross the midline (Kullander et al., 2003) ALWAYS at a spinal level. To my knowledge, nobody has ever shown hopping occurring because of supraspinal tract alteration in rodents. Moreover, unlike humans’ the CST of rodents does not directly project to motorneuron pools. Please, correct the Introduction according to these facts.

We do agree with the reviewers that mice are not a universal model of human physiology. We have modified some of the sentences about the corticospinal tract in our Introduction; however, we think that it is important to introduce some aspects of the corticospinal tract (especially with respect to DCC) as it is the focus of three figures of our manuscript, and this is an important tract impaired in human mirror movements. We have also made other significant changes to the Introduction, in line with the reviewers’ comments.

We also agree with the reviewer that hopping has not been shown to occur due to bilateral corticospinal tract projection. In most cases, it results from defective circuits in the spinal cord. However, bilaterally projecting CST can induce bilateral movements of the forelimbs, similar to a mirror movement (Serradj et al., J Neurosci 2014). We have modified our manuscript accordingly.

Results section should be adapted to fit the general line of the manuscript: that heterozygote DCC suppression produce mirror movement in humans but not in mice.

Lines 223- 227 I don’t know what is ’voluntary’ in mice. I guess that the authors should include forelimb reaching as the closest estimate of ’voluntary movements’ in mice. But forelimb show preserved normal asymmetry in the experiments.

We thank the reviewers for their comment. We have edited the manuscript to clarify what is considered voluntary motor control and voluntary locomotor control. While locomotion on a smooth surface can be achieved by subcortical and spinal circuits, voluntary motor tasks and voluntary locomotion rely on supraspinal systems as the animal is required to adapt its movements to the environment, to reach an object, or to avoid an obstacle. Thus, voluntary motor control corresponds to skilled forelimb reaching tasks and can be assessed in the cylinder test (Bretzner et al., 2008; Bretzner et al., 2010; Sparling et al., 2015). In contrast, the horizontal ladder test is used to assess voluntary locomotor control (Metz and Whishaw, 2002; Laflamme et al. 2018).

To clarify this, the Results section has been edited:

Line 236 : “In mice, rats, and cats, while locomotion on a smooth horizontal surface may rely solely on subcortical and spinal motor systems (Soblosky et al., 2001, Z’Graggen et al., 1998), the motor cortex plays an important role in the control of voluntary motor tasks such as skilled forelimb reaching (Whishaw 2000; Bretzner et al., 2008; Bretzner et al., 2010; Sparling et al., 2015), as well as skilled locomotion while the animal has to adjust its precise paw and limb trajectory to avoid obstacles (Drew et al., 1996; Metz and Whishaw 2002; Friel et al., 2007; Laflamme et al., 2019).”

Line 243: “Using a test of vertical exploration to assess skilled motor control (Figure 1A-B), ...”

Line 277: “To evaluate whether skilled forelimb locomotion might be impaired upon Dcc mutation, we also assessed mice while crossing a horizontal ladder with even-spaced rungs, a situation where there is a need for precise limb trajectories and paw placements. (Figure 1I-K).”

At most treadmill speeds, there is a mix of gaits expressed which may influence the data. Alternating gaits will have similar results in phasing plots but there are clear differences when examining the step order and duration - which are less obvious than full alternation vs full synchrony and may be expected with a weaker phenotype. Were the gaits (i.e. trot, lateral walk, out of phase walk - see Lemieux et al 2016) expressed in similar percentages by DCC heterozygous mice and wildtype mice?

As suggested by the reviewers, we have analyzed the frequency of gait occurrence according to genotype. The new Figure 7 shows a decrease in the occurrence of asymmetrical walk in Dcc mutant mice in comparison to their WT littermates at slow treadmill speed. We have included a method describing how gait analysis was performed and new results are now mentioned in the discussion, the intro, and the abstract. Subsequent figures have been reordered accordingly.

All locomotion (in vivo and in vitro) is relatively slow/low frequency locomotion. V0 and V3 neurons should be the ones affected with the DCC mutation and these have been shown to be active in a speed-dependent manner. Could there be locomotor phenotypes at other speeds? The locomotor effects of the removal of the V0V population were not seen until frequencies greater than 0.35-0.4 Hz (Talpalar et al 2013, which corresponds to 2500-2850ms in Fig 8). Such frequencies should be possible with >7.5uM NMDA. Minimally, this should be mentioned in the discussion.

As suggested by the reviewers, we have added a sentence in the discussion:

Line 524: “given the absence of changes in ... and in the coupling during fictive locomotion even at high NMDA concentrations using isolated spinal cord preparation...

Burst amplitudes are highly dependent on the seal of the suction electrode (size of the root, size of the electrode, etc). Given that this is one of the main positive findings of the study, some detail should be added to the Methods about the electrodes (were they all of a standard size or selected based on size relative to root?) and about the measurement of amplitude (if from an integrated signal, was amplitude measured from the baseline (0) or the background activity/noise).

As suggested by the reviewers, we have edited the Methods to provide more details on the neonatal fictive locomotion experiments:

Line 204: “Left and right lumbar L2 and L5 ventral roots were attached to suction electrodes designed and selected to fit the specific size of each recorded ventral root, thus ensuring a perfect seal of the suction electrode.”

Line 216: “The amplitude was measured from the baseline (0) of integrated signals.”

The role of cortex in the control of locomotion in rodents is not clear. Therefore, the authors’ assumption that cortex produces ’voluntary locomotor control’ is a bit too anthropocentric. It is known that mice, and even cats, can produce reasonable locomotion without cortex (see classic literature like Grillner’s review 1981; Skik and Orlovsky, 1966, etc.). There is no need for such a dubious claim. The authors study spontaneous motor control and locomotion.

We have better defined what we considered as “voluntary locomotion” in the manuscript. “Voluntary” or “skilled” locomotion, in situations where there is a need to avoid obstacles, is guided largely by sensory feedback. Studies in cats have shown that the motor cortex greatly contributes to the execution of gait modifications and is involved both in specifying limb trajectory and paw placement (Adkins et al., 1971, Chambers and Liu, 1957, Eidelberg and Yu, 1981, Gorska et al., 1993, Jiang and Drew, 1996, Laursen and Wiesendanger).

Lines 324-325 Can treadmill locomotion be considered ’voluntary’? The treadmill evokes or initiate locomotion but locomotor control is dominantly under spinal and maybe some subcortical structure control. Mice may ’willingly’ engage in following the treadmill or not, but the locomotion performance is nearly automatic. I don’t think that mice are able to ’voluntarily’ change their gaits like humans can eventually do. Please, eliminate ’voluntary’ where not properly applied.

As mentioned above, we have edited the manuscript to clarify what we consider voluntary (or skilled) locomotor control. Voluntary/skilled locomotion was assessed by the horizontal ladder test, placing the animal in a situation where there is a need for precise limb trajectories and paw placements.

Line 277: “To evaluate whether skilled forelimb locomotion might be impaired upon Dcc mutation, we also assessed mice while crossing a horizontal ladder with even-spaced rungs, a situation where there is a need for precise limb trajectories and paw placements. (Figure 1I-K).”

Physiological assessment of CST by electrical stimulation. Mice data is COMPLETELY different from human. Electric or TMS stimulation in humans has a short delay. Humans CST conveys directly to motor pools, while rodent CST is projected to the dorsal horn. So, pathways are different. Human motor control is highly dependent of cortex while this is not the case in other animals like cats and mice. The fact cortical stimulation produces some movement in mice hind limbs has a more complex explanation than in humans. Please, adapt the text considering these known differences. Moreover, the fact that the authors need a train of stimuli to evoke CST induction of burst on TA muscle adds complexity to the interpretation of a signal that occurs about >40 ms after the first stimulus. Therefore the physiological interpretation of this long latency measurement is not straightforward to me. Though, it’s clear that is not changed in DCC+/- animals.

Regarding the corticospinal pathways, while monosynaptic connectivity is assumed to be more important in humans and non-human primates than in other species, such as the cat, the latter is more likely to rely on propriospinal neurons conveying corticospinal inputs to motoneurons compared with primates (Nakajima et al., 2000). In rodents, corticospinal tract projections from the murine motor cortex mostly target glutamatergic neurons of lamina VII (Ueno et al. 2018), a region involved in spinal motor control and containing propriospinal interneurons.

It is difficult to compare motor-evoked potentials in humans with those in animal models because different methodologies are used. In humans, responses are evoked either with transcranial electric or magnetic stimulation using single pulses at high intensity (Merton and Morton, 1980; Reis et al., 2000). Both types of transcranial stimulation generate responses of similar amplitude and latency, at least for the corticospinal descending volley (Burke et al., 1993). In animal models, motor activity is more often evoked with intracranial microstimulation using electrodes inserted in deep layers containing cell bodies of corticospinal neurons. Intracranial stimulation is delivered with high frequency (300-350 Hz) trains of low intensity (below 75 μA, usually about 5-35 μA) biphasic pulses that generally elicit EMG responses within 10-30 ms in latency in monkeys (Asanuma and Rosen, 1972), cats (Armstrong and Drew 1985; Bretzner and Drew, 2005), and rats (Watson et al., 2018). Longer latencies (up to 50 ms) have also been reported under ketamine anesthesia (Gu and Fortier, 1996), as in the current study.

As there are no changes in the motor cortex and the corticospinal tract in mutant mice, and since the reviewers asked us to shorten the discussion, we agree that it is not necessary to further discuss this point in detail, and we have thus shortened the Discussion accordingly.

Figure 10 Some mutants produce more variance like the cited studies (Zhang et al, 2008), but other produce LESS variance (Bellardita and Kiehn, 2015). The authors should cite more widespread the highly relevant literature...

We now cite the Bellardita and Kiehn (2015) study when we introduce Figure 11:

Line 425: “Although some mutant mice can produce a more variable locomotor coordination (Zhang et al, 2008), others produce a less variable one (Bellardita and Kiehn, 2015). “

The differences that the authors see during treadmill locomotion and during in vitro locomotor-like recordings are MINIMAL. I don’t think that they reflect any highly specific change. The number of animals is not large. I wouldn’t draw tremendous conclusion from such a small sample size.

It is correct that the mouse phenotypes (increased stance phase in adult treadmill locomotion and increase in the amplitude of flexor motor output during in vitro locomotor-like activity in the neonatal isolated cord) that we observed in Dcc heterozygous mice are subtle. Nonetheless, they generated interest in performing a detailed study of locomotion in DCC heterozygous humans. In line with the observation of subtle locomotor phenotypes in Dcc heterozygous mice, recent unpublished data from our collaborator, using treadmill kinematic analysis, showed that DCC heterozygous humans also have a subtle locomotion defect. This human investigation was directly driven by our finding that mice have a subtle defect. Thus, despite being subtle, these defects might nonetheless reveal interesting areas of investigations in human pathological conditions.

Figure 11. There is no proper rationale for the use of burst deletions in this work. Which kind of information it conveys? What does it mean? The results are minimal if any and at the lowest NMDA concentration (see Talpalar and Kiehn, 2010 for proper methods and rationale if the authors want). I propose to eliminate those data from the manuscript. As such it doesn’t provide any useful information.

This was a request from the previous round of revisions. We agree with the reviewers and have removed this section from the results, as it does not provide useful information in the new manuscript.

In the discussion, the authors insist on hypothesizing that the minor changes observed in the gait in their mouse model explain altered movement in human DCC heterozygotes. I think that the small changes that the authors see have nothing to do with mirror movements or with human pathophysiology. I infer that mirror movement occur in humans because their cortical motor function is dominant compared with rodents which rather rely on subcortical and spinal networks for motor function. Please, emphasize this difference. It’s everywhere in classic motor literature.

We totally agree. Indeed, our findings support the absence of changes in the motor cortex and the corticospinal function. We have made changes to our Discussion to reflect this comment.

The changes that the authors see in the gait and locomotor-like activity are MINOR and the number of animals is small. I don’t think that it makes any sense trying to predict that they have anything to do with the robust features of human pathology in adults. Do DCC deficient humans show mirror movements since birth? I don’t know. If they don’t, then mirror movements are not innate, and may be learnt while learning motor and locomotor functions later in life. Please, shorten the discussion and focus it on the fact that mice DO NOT mimic human mirror movements.

Mild physiological mirror movements can occur in normally developing children until the age of 7 yo and are not considered pathological. Higher intensity mirror movements before the age of 7 yo are abnormal. Also, if mirror movements (even mild) persist past 7 yo, this individual is diagnosed as having mirror movements.

Nevertheless, we have shortened the Discussion as requested. Despite being subtle, the phenotypical defects that we observed might nonetheless reveal interesting future areas of investigations in human pathological conditions, as new data in human DCC heterozygous individuals from our collaborator indicate. For this reason, we would prefer to keep this element in our Discussion.

Minor issues

Page 3 Line 53 I don’t think that description of hopping in a tetrapod can be ’kangaroo-like’ provided that kangaroos have a special bipedal or tripod gait with contribution of the tail. I think that a tetrapod instance in more appropriate, rabbit-like for example.

We have corrected to rabbit-like gait.

References: The layout of the different references is inconsistent. References layout should follow the journal guidelines for references.

We have corrected this.

The conclusion in lines 381-384 (’more resilient to high NMDA concentrations ....upon loss of one Dcc allele’.) is strong given that there is no statistically significant change with concentration of drug in either wt or DCC+/-.

This is now corrected:

Line 408: “This increased motor output suggests that spinal motoneurons and/or the spinal interneuronal circuit (at least in the L2 segment) are more excitable upon bath application of NMDA in the developing spinal cord upon loss of one Dcc allele.”

Figure 6: Color coding in A-C is difficult to differentiate between 15 and 20cm/sec, given the line width of the vectors. The description in the legend (i.e. “WT(black circles)”) does not match what is shown. Are the points in A-C just at one speed but vectors at 3? The “dashed inner circles” are not dashed.

Thanks for pointing this out. We have corrected this.

Since differences in left-right coordination deficits are those most consistent with the DCC-/- mutant phenotype and the lack of deficit fits with the Rabe Bernhart study, I suggest putting the phasing (Fig 10) ahead of or integrated with Fig 8, prior to going to the subtleties.

In order to keep consistency with the presentation of the treadmill locomotion data, we prefer to keep the current organization of the neonatal locomotion.

Lines 518-522 are not clear. They should be split into two sentences and collapsed MNs should be explained.

We have modified the sentence and removed the term collapse.

Lines 522-524 - Abnormal excitability in MNs could also be abnormal drive to them.

We have corrected this:

Line 546: “However, the amplitude of the flexor-related motoneuronal activity was significantly increased in Dcc+/- spinal cords upon bath application of NMDA, suggesting that DCC plays a role in increasing the excitability of the spinal interneuronal circuit and/or motoneuronal activity.”
