# Supplementary material for: Heterozygous Dcc Mutant Mice Have a Subtle Locomotor Phenotype
Source: eNeuro. 2022 Mar 2;9(2):ENEURO.0216-18.2021. doi: 10.1523/ENEURO.0216-18.2021 (PMC8906791; doi:10.1523/ENEURO.0216-18.2021)
Supplement: Extended Data Table 1-1 — Skilled motor control in adult Dcc+/− and WT mice. Download Table 1-1, DOC file. [file enu-eN-NWR-0216-18-s01.doc]

Table 1-1

| **Data (n, mean ±SD)** | **Distribution and variance test** | **Statistic test** | **P value** |
| --- | --- | --- | --- |
| **Fig. 1A: Cylinder test:**  **First contact (%)**  WT: n=6  Left mean 56.84 ±22.87  Right mean 29.74 ±9.588  Both mean 13.42 ±16.28  DCC: n=7  Left mean 55.71 ±18.58  Right mean 28.57 ±21.93  Both mean 15.71 ±8.864 | Shapiro-Wilk :  WT N too small  Normal distribution | Mann Whitney | Left, p=0.9226  Right, p=0.6669  Both, p=0.5163 |
| **Fig. 1B: Cylinder test: Total contact (%)**  WT: n=6  Left mean 47.31 ±9.274  Right mean 36.17 ±8.985  Both mean 16.52 ±7.224  DCC: n=7  Left mean 38.10 ±13.06  Right mean 39.78 ±12.30  Both mean 22.12 ±12.44 | Shapiro-Wilk :  Not normal distribution | Mann Whitney | Left, p=0.2343  Right, p=0.5643  Both, p=0.3534 |
| **Fig. 1C: Beam locomotion: Number of steps (FL)**  6 mm and 12 mm  WT: n= 6 x 3 crossings  DCC: n= 7 x 3 crossings | Bartlett test | One-way Anova | 6 mm  WT, p=0.408  DCC, p=0.5901  12 mm  WT, p=0.9119  DCC, p=0.8565 |
| The variance intra-group was not significant, so all data were pooled | | | |
| Pooled data  WT: = 6 x 3 crossings  6 mm 15.17 ±1.295  12 mm 12.61 ±1.290  DCC: = 7 x 3 crossings  6mm 15.19 ±2.040  12mm 13.05 ±1.532 | Shapiro-Wilk test :  Normal distribution | Mann Whitney | WT vs DCC  6 mm: p= 0.8392  12 mm, p= 0.3193  **6 mm x 12 mm**  **WT: *** p/ 0.0001**  **DCC: ** p= 0.0011** |
| **Fig. 1D: Beam locomotion: Time (ms)**  6 mm and 12 mm  WT: n=6 x 3 crossings  DCC: n= 7 x 3 crossings | Bartlett test | One-way Anova  Kruskal-Wallis for DCC at 12 mm | 6 mm  WT, p=0.1792  DCC, p=0.8381  12 mm  WT, p=0.2184  DCC, p=0.4995 |
| The variance intra-group was not significant, so all data were pooled | | | |
| Pooled data  WT: n = 6 x 3 crossings  6 mm mean 7.556 ±2.595  12 mm mean 5.056 ±0.9984  DCC: n = 7 x 3 crossings  6 mm mean 7.429 ±3.091  12 mm mean 5.429 ±2.357 | Shapiro-Wilk test:  Normality (except WT at 12 mm). | Mann Whitney | WT vs DCC  6 mm, p= 0.6145  12 mm, p= 0.8249  **6 mm x 12 mm**  **WT, *** p= 0.0002**  **DCC, ** p= 0.0011** |
| **Fig. 1E: Beam locomotion: Proportion of successful/failed crossings**  WT: n = 6 x 3 crossings  6 mm  N success = 11 (61.111 %)  N error = 7 (38.889 %)  12 mm  N success = 16 (88.889 %)  N error = 2 (11.111 %)  DCC: n = 7 x 3 crossings  6 mm  N success = 9 (42.857 %)  N error = 12 (57.143 %)  12 mm  N success = 15 (71.428 %)  N error = 6 (28.571 %) | Shapiro-Wilk test:  N too small (WT n=6)  Normality (DCC 6 mm)  No normality (DCC 12mm) | Mann Whitney | WT vs DCC  6 mm, p= 0.4128  12 mm, p= 0.3771  6 mm vs 12 mm  WT, p= 0.1398  DCC, p= 0.1626 |
| **Fig. 1F: Beam locomotion: Proportion of successful/failed crossings**  WT: n = 6 x 3 crossings  6 mm  N success = 8 (44.444 %)  N error = 10 (55.556 %)  12 mm  N success = 14 (77.778 %)  N error = 4 (22.222 %)  DCC: n = 7 x 3 crossings  6 mm  N success = 14 (66.667 %)  N error = 7 (33.333 %)  12 mm  N success = 15 (71.429 %)  N error = 6 (28.571 %) | Shapiro-Wilk test:  N too small  (WT n=6)  Normality  (DCC 6 mm)  No normality  (DCC 12 mm) | Mann Whitney | WT vs DCC  6 mm, p= 0.3001  12 mm, p= 0.3938  6 mm vs 12 mm  WT, p= 0.1534  DCC, p= 0.9456 |
| **Fig. 1G: Beam locomotion: proportion of foot-slips with the forelimb (%)**  6 mm, WT n=5 DCC n= 4  12 mm, WT n= 2 DCC n=5  (1 to 3 values per mouse) | 6 mm  WT : Fisher test  DCC : Bartlett test  12 mm  WT : Fisher test  DCC : Fisher test | 6 mm  WT: Kruskal-Wallis  DCC: Anova  12 mm  WT: Kruskal-Wallis  DCC: Kruskal-Wallis | 6mm  WT, p=0.5130  DCC, p=0.6905  12mm  WT n too small  DCC, p=0.2873 |
| The variance intra-group was not significant, so all data were pooled | | | |
| Pooled data  WT 6mm  n=7, mean: 5.514 ±2.908  WT 12mm  n=2, mean: 3.869 ±0.4209  DCC 6mm  n= 12, mean: 6.106 ±2.856  DCC 12mm  n= 6, mean: 6.804 ±4.814 | Shapiro-Wilk test :  N too small  (WT and DCC 12 mm)  No normality  (WT 6mm)  Normality (DCC 6mm) | Mann Whitney | WT vs DCC  6 mm, p=0.8322  12 mm: n too small for Mann-Whitney  6 mm vs 12 mm  WT, n too small  DCC, p= 0.8144 |
| **Fig. 1H: Beam locomotion:**  **proportion of foot-slips with the hindlimb (%)**  6 mm WT n= 4 DCC n=6  12 mm WT n=2 DCC n= 4  (1 to 3 values per mouse) | 6 mm  Bartlett test  12 mm  WT : too many zeroes  DCC : Bartlett | 6 mm  WT: Kruskal-Wallis  DCC: One-Way Anova  12 mm  WT: Kruskal-Wallis  DCC: One-way Anova | 6 mm  WT, p= 0.0648  DCC, p= 0.3963  12 mm  WT, p= 0.3679  DCC, p= 0.3965 |
| The variance intra-group was not significant, so all data were pooled | | | |
| Pooled data  WT 6 mm  n= 10 mean: 5.989 ±3.126  WT 12 mm  n= 4 mean: 4.101 ±0.3325  DCC 6 mm  n= 7 mean: 7.268 ±3.588  DCC 12 mm  n= 6 mean: 4.556 ±1.422 | Shapiro-Wilk test :  Normality 6 mm  N too small 12 mm | Mann Whitney | WT vs DCC  6 mm, P=0.4336  12 mm, P=1.0000  6 mm vs 12 mm  WT, p= 0.9433  DCC, p= 0.2208 |
| **Fig. 1I: Ladder locomotion Number of steps (LFL)**  WT: n= 6 x 3 crossings  DCC: n= 7 x 3 crossings | Bartlett | One-way Anova | WT, p=0.9734  DCC, p=0.5711 |
| The variance intra-group was not significant, so all data were pooled | | | |
| Pooled data  WT: n= 6 x 3 crossings  mean: 5.778 ±1.517  DCC: n= 7 x 3 crossings  Mean: 5.762 ±1.136 | Shapiro-Wilk test:  Not normal distribution | Mann Whitney | WT vs DCC, p= 0.8840 |
| **Fig. 1J: Proportion of successful/failed crossings**  WT: n = 6 x 3 crossings  N success = 4 (22.222 %)  N failed = 14 (77.778 %)  DCC: n = 7 x 3 crossings  N success = 2 (9.5238 %)  N failed = 19 (90.476 %) | Shapiro-Wilk test:  N too small (WT n=6)  No normality (DCC) | Mann Whitney | WT vs DCC, p= 0.6614 |
| **Fig. 1K: Ladder locomotion Errors Forelimb (%)**  WT: n=6  DCC: n=7  (1 to 3 values per mouse) | WT: too many zeroes  DCC: Bartlett | WT: Kruskal-Wallis  DCC: One-way Anova | WT, p=0.0673  DCC, p=0.73286 |
| The variance intra-group was not significant, so all data were pooled | | | |
| Pooled data  WT: n=14  Mean: 14.70 ±9.764  DCC: n=19  Mean: 12.98 ±7.417 | Not normal distribution | Mann Whitney | WT vs DCC, p = 0.4091 |

Table 4-1

| Data (n, mean, SD) | Distribution and variance test | Statistic test | P value |
| --- | --- | --- | --- |
| Fig. 4A Step cycle duration (s)  WT vs. DCC | | | |
| 15cm/s  WT n=8 mean 0.3403 ±0.05769  DCC n=10 mean 0.3620 ±0.03387 | 15cm/s  Test: Shapiro-Wilk  WT= Normal  DCC= Normal | Unpaired T test with Welch’s correction | p= 0.3673 |
| 20cm/s  WT n=8 mean 0.2843 ±0.03241  DCC n=10 mean 0.2951 ±0.01984 | 20cm/s  Test: Shapiro-Wilk  WT= Normal  DCC= Normal | Unpaired T test with Welch’s correction | p=0.4291 |
| 30 cm/s  WT n=8 mean 0.2267 ±0.01637  DCC n=9 mean 0.2463 ±0.04247 | 30cm/s  Test: Shapiro-Wilk  WT= Normal  DCC= Not normal | Unpaired  Mann Whitney | p=0.3213 |
| Fig. 4B Swing  WT vs. DCC | | | |
| 15cm/s  WT n=8 mean 0.1265 ±0.02141  DCC n=10 mean 0.1206 ±0.01643 | 15cm/s  Test: Shapiro-Wilk  WT= Normal  DCC= Normal | Unpaired T test with Welch’s correction | P= 0.5314 |
| 20cm/s  WT n=8 mean 0.1247 ±0.02274  DCC n=10 mean 0.1134 ±0.006628 | 20cm/s  Test: Shapiro-Wilk  WT= Normal  DCC= Normal | Unpaired T test with Welch’s correction | P= 0.2127 |
| 30 cm/s  WT n=8 mean 0.1056 ±0.01409  DCC n=9 mean 0.1092 ±0.01560 | 30cm/s  Test: Shapiro-Wilk  WT= Not normal  DCC= Normal | Unpaired  Mann Whitney | P= 0.8686 |
| Fig. 4C Stance  WT vs. DCC | | | |
| 15cm/s  WT n=8 mean 0.2138 ±0.04138  DCC n=10 mean 0.2414 ±0.02408 | 15cm/s  Test: Shapiro-Wilk  WT= Normal  DCC= Normal | Unpaired T test with Welch’s correction | P=0.1233 |
| 20cm/s  WT n=8 mean 0.1597 ±0.02826  DCC n=10 mean 0.1816 ±0.01670 | 20cm/s  Test: Shapiro-Wilk  WT= Normal  DCC= Normal | Unpaired T test with Welch’s correction | P= 0.0784 |
| 30 cm/s  WT n=8 mean 0.1211 ±0.01526  DCC n=9 mean 0.1371 ±0.03134 | 30cm/s  Test: Shapiro-Wilk  WT= normal  DCC= Not normal | Unpaired  Mann Whitney | P=0.3088 |
| Fig 4D Duty cycle  WT vs. DCC | | | |
| 15cm/s  WT n=8 mean 61.62 ± 4.020  DCC n=10 mean 66.28 ±2.845 | 15cm/s  Test: Shapiro-Wilk  WT= Not normal  DCC= Normal | Unpaired  Mann Whitney | **p=0.0085 |
| 20cm/s  WT n=8 mean 55.94 ± 6.485  DCC n=10 mean 61.46 ±2.145 | 20cm/s  Test: Shapiro-Wilk  WT= Normal  DCC= Normal | Unpaired T test with Welch’s correction | *p=0.0490 |
| 30 cm/s  WT n=8 mean 53.40 ±5.919  DCC n=9 mean 55.32 ±4.035 | 30cm/s  Test: Shapiro-Wilk  WT= normal  DCC= normal | Unpaired T test with Welch’s correction | P= 0.4541 |

VARIABILITY

| Fig. 4E Step cycle duration (s) CV % WT vs. DCC | | | |
| --- | --- | --- | --- |
| 15cm/s  WT n=8 mean 20.76 ± 9.502  DCC n=10 mean 15.65 ±5.484 | 15cm/s  Test: Shapiro-Wilk  WT= Not normal  DCC= Normal | Unpaired T test with Welch’s correction | P=0.2048 |
| 20cm/s  WT n=8 mean 9.625 ± 2.960  DCC n=10 mean 10.23 ±2.640 | 20cm/s  Test: Shapiro-Wilk  WT= Normal  DCC= Normal | Unpaired T test with Welch’s correction | P=0.6584 |
| 30 cm/s  WT n=8 mean 10.29 ± 3.410  DCC n=9 mean 9.323 ±3.328 | 30cm/s  Test: Shapiro-Wilk  WT= normal  DCC= not normal | Unpaired  Mann Whitney | ¨p=0.4234 |
| Fig 4F Swing CV %  WT vs. DCC | | | |
| 15cm/s  WT n=8 mean 24.47 ± 8.015  DCC n=10 mean 22.45 ± 8.873 | 15cm/s  Test: Shapiro-Wilk  WT= Not normal  DCC= Normal | Unpaired T test with Welch’s correction | P=0.6192 |
| 20cm/s  WT n=8 mean 15.11 ± 6.876  DCC n=10 mean 16.00 ±3.344 | 20cm/s  Test: Shapiro-Wilk  WT= Normal  DCC= Normal | Unpaired T test with Welch’s correction | P=0.7437 |
| 30 cm/s  WT n=8 mean 13.21 ± 4.967  DCC n=9 mean 12.49 ± 4.500 | 30cm/s  Test: Shapiro-Wilk  WT= normal  DCC= normal | Unpaired T test with Welch’s correction | P=0.7583 |
| Fig 4G Stance CV %  WT vs. DCC | | | |
| 15cm/s  WT n=8 mean 32.64 ± 12.05  DCC n=10 mean 22.36 ±9.682 | 15cm/s  Test: Shapiro-Wilk  WT= Not normal  DCC= Normal | Unpaired T test with Welch’s correction | P=0.0713 |
| 20cm/s  WT n=8 mean 17.81 ± 4.373  DCC n=10 mean 13.43 ±3.591 | 20cm/s  Test: Shapiro-Wilk  WT= Normal  DCC= Normal | Unpaired T test with Welch’s correction | *p= 0.0392 |
| 30 cm/s  WT n=8 mean 13.87 ± 4.486  DCC n=9 mean 13.45 ±5.398 | 30cm/s  Test: Shapiro-Wilk  WT= normal  DCC= normal | Unpaired T test with Welch’s correction | P=0.8620 |
| Fig 4H Duty cycle CV %  WT vs. DCC % | | | |
| 15cm/s  WT n=8 mean 16.37 ±4.960  DCC n=10 mean 11.59 ±4.662 | 15cm/s  Test: Shapiro-Wilk  WT= Not normal  DCC= Normal | Unpaired T test with Welch’s correction | *p=0.0420 |
| 20cm/s  WT n=8 mean 12.30 ± 5.556  DCC n=10 mean 8.003 ±2.124 | 20cm/s  Test: Shapiro-Wilk  WT= Normal  DCC= Normal | Unpaired T test with Welch’s correction | P=0.0696 |
| 30 cm/s  WT n=8 mean 7.892 ± 1.950  DCC n=9 mean 8.158 ±3.528 | 30cm/s  Test: Shapiro-Wilk  WT= normal  DCC= not normal | Unpaired  Mann Whitney | P= 0.8485 |

**Table 9-1**

| **Data (n, mean SD)** | **Distribution and variance test** | **Statistic test** | **P value** |
| --- | --- | --- | --- |
| **Fig. 9C Step cycle duration (ms) L2**  **WT vs. DCC**  **2.5 uM**  WT n=4 mean 6062 ±1864  DCC n=7 mean 6819 ±2136  **5 uM**  WT n=4 mean 4760 ±844.3  DCC n=7 mean 4967 ±732.6  **7.5 uM**  WT n=4 mean 4072 ±179.7  DCC n=7 mean 3897 ±840.9 | WT: N too small  DCC: Normality (except 7.5 uM) | Mann Whitney | **WT vs DCC**  **2.5 uM**  P= 0.6485  **5 uM**  P= 0.9273  **7.5 uM**  p= 0.6485 |
| **Fig. 9D Burst duration (ms) L2**  **WT vs DCC**  **2.5 uM**  WT n=4 mean 3697 ±923.7  DCC n=7 mean 4441 ±1778  **5 uM**  WT n=4 mean 3305 ±756.3  DCC n=7 mean 3558 ±785.0  **7.5 uM**  WT n=4 mean 3230 ±217.3  DCC n=7 mean 2884 ±787.5 | WT: N too small  DCC: Normality | Mann Whitney | **WT vs DCC**  **2.5 uM**  P=0.4121  **5 uM**  P=0.7879  **7.5 uM**  p= 0.2303 |
| **Fig. 9E Duty Cycle L2**  **WT vs DCC**  **2.5 uM**  WT n=4 mean 3697 ±923.7  DCC n=7 mean 4441 ±1778  **5 uM**  WT n=4 mean 3305 ±756.3  DCC n=7 mean 3558 ±785.0  **7.5 uM**  WT n=4 mean 3230 ±217.3  DCC n=7 mean 2884 ±787.5 | WT: N too small  DCC: Normality | Mann Whitney | **WT vs DCC**  **2.5 uM**  p= 0.5273  **5 uM**  p= 0.6485  **7.5 uM**  p= 0.1091 |

| **Fig. 9F Amplitude (mV) L2**  **WT vs DCC**  **2.5uM**  WT n=4 mean 0.02791 ±0.02753  DCC n=7 mean 0.07089 ±0.03342  **5uM**  WT n=4 mean 0.03404 ±0.03314  DCC n=7 mean 0.08595 ±0.03578  **7.5uM**  WT n=4 mean 0.02237 ±0.01028  DCC n=7 mean 0.05240 ±0.02295 | WT : N too small  DCC : Normality | Mann Whitney | **WT vs DCC**  **2.5 uM**  *p= 0.0424  **5 uM**  *p= 0.0424  **7.5 uM**  *p= 0.0242 |
| --- | --- | --- | --- |

| **Fig. 9G Step cycle duration (ms) L5**  **WT vs DCC**  **2.5 uM**  WT n=4 mean 6439 ±1534  DCC n=7 mean 7889 2877  **5 uM**  WT n=4 mean 4687 ±944.8  DCC n=7 mean 4802 ±652.4  **7.5 uM**  WT n=4 mean 4158 ±300.7  DCC n=7 mean 3846 ±902.3 | WT : N too small  DCC : Normality | Mann Whitney | **WT vs DCC**  **2.5 uM**  p= 0.5273  **5 uM**  p= 0.7879  **7.5 uM**  p= 0.2303 |
| --- | --- | --- | --- |
| **Fig. 9H Burst duration (ms) L5**  **WT vs DCC**  **2.5 uM**  **WT** n=4 mean 3897 ±1289;  **DCC** n= 7 mean 4496 ±1356  **5 uM**  WT n=4 mean 3858 ±644.2;  DCC n=7 mean 3536 ±559.4  **7.5 uM**  WT n= 4 mean 3356 656.0  DCC n=7 mean 3217 ±869.3 | WT : N too small  DCC : Normality | Mann Whitney | **WT vs DCC**  **2.5 uM**  p = 0.3152  **5 uM**  p= 0.4121  **7.5 uM**  p= 0.5273 |

| **Fig. 9I Burst duration (ms) L5**  **WT vs DCC**  **2.5 uM**  **WT** n=4 mean 3897 ±1289;  **DCC** n= 7 mean 4496 ±1356  **5 uM**  WT n=4 mean 3858 ±644.2;  DCC n=7 mean 3536 ±559.4  **7.5 uM**  WT n= 4 mean 3356 656.0  DCC n=7 mean 3217 ±869.3 | WT : N too small  DCC : Normality | Mann Whitney | **WT vs DCC**  **2.5 uM**  p= 0.7879  **5 uM**  p= 0.1091  **7.5 uM**  p= 0.4121 |
| --- | --- | --- | --- |
| **Fig. 9J Burst duration (ms) L5**  **WT vs DCC**  **2.5 uM**  **WT** n=4 mean 3897 ±1289;  **DCC** n= 7 mean 4496 ±1356  **5 uM**  WT n=4 mean 3858 ±644.2;  DCC n=7 mean 3536 ±559.4  **7.5 uM**  WT n= 4 mean 3356 656.0  DCC n=7 mean 3217 ±869.3 | WT : N too small  DCC : Normality | Mann Whitney | **WT vs DCC**  **2.5 uM**  p= 0.4121  **5 uM**  p= 0.4121  **7.5 uM**  p= 1.000 |

**Table 10-1**

| **Data (n, mean SD)** | **Normality test:**  **Shapiro-Wilk test** | **Type of test** | **P value** |
| --- | --- | --- | --- |
| **Fig. 10A Cycle duration CV (%) L2**  **WT vs DCC**  **2.5 uM**  WT n=4 mean 44.11 ±8.470  DCC n=7 mean 67.87 ±25.43  **5 uM**  WT n=4 mean 27.85 ±6.747  DCC n=7 mean 33.77 ±15.84  **7.5 uM**  WT n=4 mean 18.35 ±13.70  DCC n=7 mean 20.07 ±8.852 | WT n too small  DCC : Normality | Mann Whitney | **2.5 uM**  Yes * p= 0.0242  **5 uM**  Ns p= 1.0000  **7.5 uM**  Ns p= 0.6485 |
| **Fig. 10B Burst duration CV (%) L2**  **WT vs DCC**  **2.5 uM**  WT n=4 mean 50.79 ±8.802  DCC n=7 mean 71.93 ±32.65  **5 uM**  WT n=4 mean 31.66 ±13.07  DCC n=7 mean 46.45 ±21.60  **7.5 uM**  WT n=4 mean 17.78 ±5.497  DCC n=7 mean 24.23 ±8.320 | WT : N too small  DCC : Normality  (except 5 uM) | Mann Whitney | **2.5 uM**  Ns p= 0.2303  **5 uM**  Ns p= 0.3152  **7.5 uM**  Ns p= 0.1636 |
| **Fig. 10C Duty Cycle CV (%) L2**  **WT VS DCC**  **2.5 uM**  WT n=4 mean 25.67 ±7.335  DCC n=7 mean 26.55 ±8.701  **5 uM**  WT n=4 mean 18.95 ±6.571  DCC n=7 mean 18.68 ±9.186  **7.5 uM**  WT n=4 mean 9.688 ±2.183  DCC n=7 mean 13.96 ±7.534 | WT : N too small  DCC : Normality (except 7.5 uM) | Mann Whitney | **2.5 uM**  Ns p = 1.0000  **5 uM**  ns p= 0.9273  **7.5 uM**  ns p= 0.4121 |
| **Fig. 10D Amplitude CV (%) L2**  **WT vs DCC**  **2.5 uM**  WT n=4 mean 26.40 ±2.659  DCC n=7 mean 26.58 ±6.449  **5 uM**  WT n=4 mean 23.08 ±11.67  DCC n=7 mean 19.61 ±5.911  **7.5 uM**  WT n=4 mean 21.76 ±1.422  DCC n=7 mean 25.37 ±6.355 | WT : N too small  DCC : Normality | Mann Whitney | **2.5 uM**  Ns p= 0.6435  **5 uM**  Ns p= 0.9273  **7.5 uM**  Ns p= 0.3152 |
| **Fig. 10E Cycle duration CV (%) L5**  **WT vs DCC**  **2.5uM**  WT n=4 mean 61.02 ±15.18  DCC n=7 mean 70.82 ±19.76  **5 uM**  WT n=4 mean 25.19 ±5.914  DCC n=7 mean 31.54 ±16.71  **7.5 uM**  WT n=4 mean 23.76 ±12.49  DCC n=7 mean 22.11 ±10.72 | WT : N too small  DCC : Normality | Mann Whitney | **2.5 uM**  Ns p= 0.4121  **5 uM**  Ns p= 0.7879  **7.5 uM**  Ns p= 0.9273 |
| **Fig. 10F Burst duration CV (%) L5**  **WT vs DCC**  **2.5 uM**  WT n=4 mean 57,16 ±15.06;  DCC n=7 mean 67.09 ±12.04  **5 uM**  WT n=4 mean 27.52 ±5.988;  DCC n=7 mean 32.09 ±15.66  **7.5 uM**  WT n= 4 mean 28.61 ±13.29  DCC n=7 mean 24.12 ±12.17 | WT N too small  DCC : Normality | Mann Whitney | **2.5 uM**  Ns p= 0.6485  **5 uM**  Ns p= 0.6485  **7.5 uM**  Ns p= 0.6485 |
| **Fig. 10G Duty Cycle CV (%) L5**  **WT vs DCC**  **2.5 uM**  WT n=4 mean 25.64 ±9.870  DCC n=7 mean 31.45 ±7.472  **5 uM**  WT n=4 mean 7.258 ±1.421  DCC n=7 mean 12.88 ±4.200  **7.5 uM**  WT n= 4 mean 11.73 ±8.755  DCC n=7 mean 8.250 ±1.791 | WT : N too small  DCC : Normality | Mann Whitney | **2.5 uM**  Ns p= 0.5273  **5 uM**  Ns p= 0.0727  **7.5 uM**  Ns p= 1.0000 |
| **Fig. 10H Amplitude CV (%) L5**  **WT vs DCC**  **2.5 uM**  WT n=4 mean 39.04 ±8.142  DCC n=7 mean 47.71 ±7.163  **5 uM**  WT n=4 mean 30.12 ±5.471  DCC n=7 mean31.39 ±7.282  **7.5 uM**  WT n=4 mean 28.29 ±1.361  DCC n=7 mean 28.00 3.023 | WT : N too small  DCC : Normality | Mann Whitney | **2.5 uM**  Ns p= 0.1636  **5 uM**  Ns p= 0.7879  **7.5 uM**  Ns p= 0.9273 |

Table 11-1

Rayleigh test for the significance of averaged vector (Table B32 from Zar, 1974)

**2.5 µM**

| ID | rL2-rL5 | iL2-rL2 | lL5 - rL5 | iL2 - lL5 | rL2- lL5 | lL2 - rL5 |
| --- | --- | --- | --- | --- | --- | --- |
| 'DCC_HE_0014_5' | n.s. | ** | n.s. | n.s. | ** | ** |
| 'DCC_HE_0018_5' | * | *** | n.s. | *** | *** | *** |
| 'DCC_HE_0019_5' | *** | *** | NaN | NaN | NaN | *** |
| 'DCC_HE_0020_5' | *** | *** | n.s. | ** | ** | *** |
| 'DCC_HE_0024_5' | *** | ** | n.s. | *** | n.s | *** |
| 'DCC_HE_0028_5' | *** | ** | NaN | NaN | NaN | * |
| 'DCC_HE_0029_5' | *** | * | NaN | NaN | NaN | * |
| 'DCC_WT_0017_5' | *** | *** | n.s. | *** | ** | *** |
| 'DCC_WT_0021_5' | *** | *** | *** | *** | *** | *** |
| 'DCC_WT_0023_5' | *** | ** | n.s. | *** | *** | *** |
| 'DCC_WT_0025_5' | *** | *** | NaN | NaN | NaN | *** |

**5 µM**

| ID | rL2-rL5 | iL2-rL2 | lL5 - rL5 | iL2 - lL5 | rL2- lL5 | lL2 - rL5 |
| --- | --- | --- | --- | --- | --- | --- |
| 'DCC_HE_0014_5' | *** | *** | *** | *** | *** | *** |
| 'DCC_HE_0018_5' | *** | *** | *** | *** | *** | *** |
| 'DCC_HE_0019_5' | *** | *** | NaN | NaN | NaN | *** |
| 'DCC_HE_0020_5' | * | *** | n.s | ** | *** | *** |
| 'DCC_HE_0024_5' | ** | *** | ** | *** | *** | *** |
| 'DCC_HE_0028_5' | *** | *** | NaN | NaN | NaN | *** |
| 'DCC_HE_0029_5' | *** | *** | NaN | NaN | NaN | *** |
| 'DCC_WT_0017_5' | *** | *** | *** | *** | *** | *** |
| 'DCC_WT_0021_5' | *** | *** | *** | *** | *** | *** |
| 'DCC_WT_0023_5' | *** | *** | n.s | ** | *** | *** |
| 'DCC_WT_0025_5' | *** | *** | NaN | NaN | NaN | *** |

**7 µM**

| ID | rL2-rL5 | iL2-rL2 | lL5 - rL5 | iL2 - lL5 | rL2- lL5 | lL2 - rL5 |
| --- | --- | --- | --- | --- | --- | --- |
| 'DCC_HE_0014_5' | *** | *** | *** | *** | *** | *** |
| 'DCC_HE_0018_5' | *** | *** | *** | *** | *** | *** |
| 'DCC_HE_0019_5' | *** | *** | NaN | NaN | NaN | *** |
| 'DCC_HE_0020_5' | *** | *** | *** | *** | *** | *** |
| 'DCC_HE_0024_5' | *** | *** | *** | *** | *** | *** |
| 'DCC_HE_0028_5' | *** | *** | NaN | NaN | NaN | *** |
| 'DCC_HE_0029_5' | *** | *** | NaN | NaN | NaN | *** |
| 'DCC_WT_0017_5' | *** | *** | *** | *** | *** | *** |
| 'DCC_WT_0021_5' | *** | *** | *** | *** | *** | *** |
| 'DCC_WT_0023_5' | *** | *** | *** | *** | *** | *** |
| 'DCC_WT_0025_5' | *** | *** | NaN | NaN | NaN | *** |

However, averaged vectors are not uniformly distributed (p>0.5) and we cannot use a Watson-William test

p-values for Mann-Whitney test on Rayleigh values

|  | 2.5 µM | 5 µM | 7.5 µM |
| --- | --- | --- | --- |
| L2 left-right | 0.5273 | 0.5273 | 0.1091 |
| L5 left-right | 0.6286 | 0.6286 | 0.8571 |
| L2-L5 right | 0.2303 | 0.4121 | 0.6485 |
| L2-L5 left | 0.4000 | 0.6286 | 0.8571 |

Proportion of significant coupling

|  |  | 2.5 µM | 5 µM | 7.5 µM |
| --- | --- | --- | --- | --- |
| L2 left-right | WT n=4 | 100 % | 100 % | 100 % (4/4) |
|  | DCC+/- n=7 | 100 % | 100 % | 100 % (7/7) |
| L5 left-right | WT n=3 | 33.33 % (1/3) | 66.66 % (2/3) | 100 % (3/3) |
|  | DCC+/- n=4 | 0 % (0/4) | 75 % (3/4) | 100 % (4/4) |
| L2-L5 right | WT n=4 | 100% | 100 % | 100 % (4/4) |
|  | DCC+/- n=7 | 85.7 % (6/7) | 100 % | 100 % (7/7) |
| L2-L5 left | WT n=3 | 100 % | 100 % | 100 % (3/3) |
|  | DCC+/- n=4 | 75 % (3/4) | 100 % | 100 % (4/4) |

Watson-William, WT vs. DCC +/-

|  | 2.5 µM | 5 µM | 7.5 µM |
| --- | --- | --- | --- |
| L2 left-right | 0.742 | 0.890 | 0.973 |
| L5 left-right | 0.880 | 0.954 | 0.582 |
| L2-L5 right | 0.705 | 0.840 | 0.894 |
